# A Tel1–Rad9 axis links DNA double-strand break end-tethering to repair pathway choice

**DOI:** 10.1093/nar/gkag919

**Published:** 2026-09-18

**Authors:** Carlo Rinaldi, Federica Pagnotta, Renata Tisi, Maria Pia Longhese

**Affiliations:** Dipartimento di Biotecnologie e Bioscienze, Università degli Studi di Milano-Bicocca, 20126 Milano, Italy; Dipartimento di Biotecnologie e Bioscienze, Università degli Studi di Milano-Bicocca, 20126 Milano, Italy; Dipartimento di Biotecnologie e Bioscienze, Università degli Studi di Milano-Bicocca, 20126 Milano, Italy; Dipartimento di Biotecnologie e Bioscienze, Università degli Studi di Milano-Bicocca, 20126 Milano, Italy

## Abstract

In eukaryotes, DNA double-strand breaks (DSBs) activate Tel1/ATM- and Mec1/ATR-dependent checkpoint signaling and can be repaired by homologous recombination (HR), which requires DNA end resection, typically initiated by the MRX/MRN complex. In budding yeast, Sae2/CtIP promotes MRX-dependent end processing, limits Tel1–Rad53 signaling, and contributes to DSB end-tethering. Here, we identify a Tel1 FAT-domain variant, Tel1-400, that increases the DNA damage resistance of *sae2*Δ cells by suppressing their DSB end-tethering defect through Rad9 phosphorylation-dependent oligomerization. Tel1-400 accumulates more strongly at DSBs than wild-type Tel1 and enhances DNA-damage-induced Rad9 phosphorylation. An oligomerization-defective *rad9* phosphosite mutant prevents Tel1-400 from improving DSB end-tethering in *sae2*Δ cells, whereas chemically induced Rad9 dimerization restores this ability. Rad9 also supports DSB end-tethering in Sae2-proficient cells, as the same *rad9* phosphosite mutant increases end separation in an otherwise wild-type background. Functionally, Tel1-400 shifts repair in *sae2*Δ cells toward strand-invasion-dependent HR while reducing single-strand annealing, and this repair-pathway bias requires Rad9 oligomerization. Together, these findings identify a Tel1–Rad9 pathway in which increased Tel1 association at DNA breaks enhances Rad9 phosphorylation and self-assembly, thereby promoting DSB end-tethering and influencing recombination pathway choice.

## Graphical Abstract

**Graphical Abstract:**
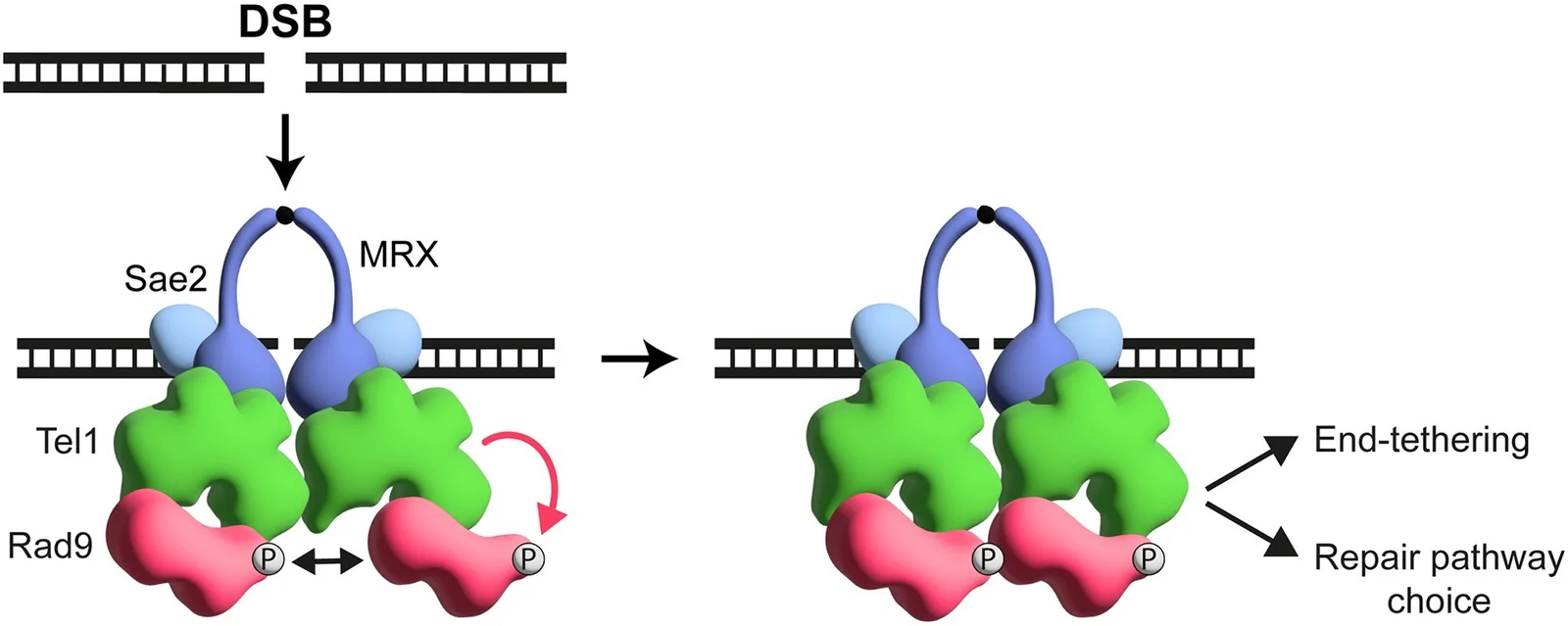

## Introduction

DNA double-strand breaks (DSBs) are highly cytotoxic lesions that, if left unrepaired or misrepaired, can lead to genome instability and chromosome rearrangements. DSBs can be repaired either by non-homologous end joining (NHEJ), which directly ligates DNA ends, or by homologous recombination (HR), which uses an intact homologous DNA molecule as a repair template [1, 2]. When a DSB arises between direct repeated sequences, it can also be repaired by single-strand annealing (SSA), a mutagenic homology-directed pathway in which complementary repeats anneal to one another, resulting in deletion of the intervening DNA together with one of the repeats [1].

A prerequisite for HR is 5′–3′ DNA end resection, which generates 3′-ended ssDNA tails competent for strand invasion [3]. In eukaryotes, resection proceeds through two major steps: the Mre11–Rad50–Xrs2/NBS1 (MRX/MRN) complex, together with Sae2/CtIP, initiates processing by clipping the 5′-terminated strands near the DNA ends, whereas long-range resection is carried out by the Exo1 and Dna2 nucleases, the latter acting with the Sgs1 helicase [4–7]. Sae2 stimulates the Mre11 endonuclease activity within the MRX complex, and this stimulation requires CDK-dependent Sae2 phosphorylation, thereby restricting DSB resection to the S and G2 phases of the cell cycle [8–10].

DSB repair is coupled to cell-cycle progression through activation of the DNA damage checkpoint, in which the apical kinases Tel1/ATM and Mec1/ATR transmit DNA damage signals to the Rad53 transduction kinase through checkpoint mediators such as budding yeast Rad9 [11]. Upon genotoxic stress, Mec1 and/or Tel1 phosphorylate Rad9 on multiple [S/T]Q sites within the SQ/TQ cluster domain (SCD), generating docking sites for the Forkhead-associated (FHA) domains of Rad53 and thereby promoting stable Rad9–Rad53 association [12–16]. Once recruited to Rad9, Rad53 is further phosphorylated by Mec1/Tel1 and undergoes trans-autophosphorylation, a step required for full kinase activation [16, 17].

Full Rad53 activation requires the ability of Rad9 to assemble into higher-order oligomers, which increase the local concentration of Rad53 and allow *trans*-autophosphorylation [18]. Rad9 oligomerization depends on its C-terminal BRCT repeats and on Mec1/Tel1-dependent phosphorylation of the SCD. Mechanistically, Rad9 assembles through intermolecular BRCT–SCD interactions, in which the BRCT domains of one Rad9 molecule bind the phosphorylated SCD of another [19]. Consistently, phosphosite mutations such as *rad9-7xA*, in which key SCD residues (T390, T398, T410, T427, S435, and T457), together with T603, are converted to alanine, disrupt BRCT–SCD binding and impair Rad9 oligomerization [19]. These mutations do not appear to affect overall Rad9 association with damaged chromatin; rather, failure to build or maintain BRCT–SCD-dependent assemblies impairs checkpoint maintenance [19].

In addition to stimulating Mre11-dependent endonucleolytic processing, Sae2 contributes to limiting Rad53 checkpoint signaling. Cells lacking Sae2 are more sensitive to DNA-damaging agents than *mre11* nuclease-dead mutants [20, 21], indicating that Sae2 also plays roles in the DNA damage response that are independent of Mre11 nuclease activity. Loss of Sae2 enhances Tel1 signaling by increasing MRX and Tel1 persistence at DSB ends [22–24]. Persistent MRX–Tel1 signaling in *sae2*Δ cells is accompanied by hyperactivation of the transducer kinase Rad53, leading to permanent cell-cycle arrest [22, 24]. Importantly, *mre11* alleles that reduce MRX binding at DSBs restore the DNA damage resistance of *sae2*Δ cells [25–27]. Similarly, compromising Tel1 function, either by reducing its association with DSBs or by lowering its kinase activity, suppresses the DNA damage sensitivity of *sae2*Δ cells [28]. Suppression is also observed when Rad53 signaling is attenuated by deleting *RAD9*, weakening Rad9–Rad53 interaction, or impairing Rad53 kinase activity [21, 28, 29]. Because Rad9 limits Sgs1 association with DSBs [20, 28, 29] and Rad53 can phosphorylate and inhibit Exo1 [30], elevated checkpoint signaling in *sae2*Δ cells also contributes to the resection defect, in addition to the loss of Sae2-dependent stimulation of Mre11 processing. Thus, the DNA-damage hypersensitivity of *sae2*Δ cells reflects a combined failure to promote proper DNA-end processing and to downregulate MRX/Tel1- and Rad53-dependent checkpoint activities.

In addition to its roles in DNA-end processing and checkpoint regulation, Sae2 contributes to maintaining the two ends of a DSB in close proximity [31, 32]. Loss of this end-tethering activity is likely to contribute to the DNA damage sensitivity of *sae2*Δ cells, as genetic conditions that reduce end separation, such as specific *mre11* or *ku70* alleles, also partially alleviate *sae2*Δ genotoxin sensitivity [27, 33]. These findings support the view that defective DSB-end architecture is an additional, functionally important component of the *sae2*Δ phenotype.

Here, we characterize a Tel1 mutant, Tel1-400, that suppresses *sae2*Δ genotoxin sensitivity without reducing checkpoint signaling or restoring DSB resection. Instead, Tel1-400 improves DSB end-tethering in *sae2*Δ cells, and this effect requires Rad9 phosphorylation-dependent oligomerization. Rad9 also contributes to DSB end-tethering independently of Sae2 loss, as both *RAD9* deletion and an oligomerization-defective *rad9* phosphosite mutant increase end separation in otherwise wild-type cells. Consistent with an architectural role for the Tel1–Rad9 axis at DSBs, *tel1-400* shifts recombination outcomes toward strand-invasion-dependent HR and away from SSA through phosphorylation-dependent Rad9 oligomerization. Together, our results support a model in which Tel1 promotes a Rad9 assembly state that helps maintain DSB-end proximity and influences the choice between distinct homology-directed repair pathways.

## Materials and methods

### Yeast strains and media

*Saccharomyces cerevisiae* was used as the experimental model. Strain genotypes are listed in Supplementary Table S1. Strains JKM139, used to detect DSB resection, YMV45, used to detect DSB repair by SSA, and tGI354, used to detect ectopic recombination, were kindly provided by J. Haber (Brandeis University, Waltham, USA). Strain YJK40.6, used to detect DSB end-tethering, was kindly provided by D.P. Toczyski (University of California, San Francisco, USA). *rad9-7xA* and the *rad9-*Δ*BRCT::FKBP* chimeric alleles were kindly provided by F. Lazzaro (University of Milan, Milan, Italy). Cells were grown in YEP medium (1% yeast extract and 2% bactopeptone) supplemented with 2% glucose (YEPD), 2% raffinose (YEPR), or 2% raffinose and 3% galactose (YEPRG). Unless otherwise indicated, yeast growth experiments were performed at 25°C. Gene disruptions and tag fusions were generated by one-step PCR and standard yeast transformation procedures. Primers used for disruptions and gene tagging are listed in Supplementary Table S2.

### Western blotting

Protein extracts for western blot analysis were prepared by trichloroacetic acid (TCA) precipitation. Frozen cell pellets were resuspended in 100 μl of 20% TCA. After the addition of acid-washed glass beads, the samples were vortexed for 10 min. The beads were washed twice with 200 μl of 5% TCA and the extract was collected in a new tube. The crude extract was centrifuged at 850 × *g* for 10 min to pellet the precipitated proteins. TCA was removed, and pellets were resuspended in 70 μl of 6 × Laemmli buffer (60 mM Tris–HCl pH 6.8, 2% SDS, 10% glycerol, 100 mM DTT, and 0.2% bromophenol blue) and 30 μl of 1 M Tris base. Prior to loading, samples were boiled and centrifuged at 850 × *g* for 10 min. The solubilized proteins in the supernatant were separated by electrophoresis on 10% SDS–polyacrylamide gels. Rad53 was detected with an anti-Rad53 polyclonal antibody (Abcam ab104232, 1:2000). HA-tagged proteins were detected with the anti-HA monoclonal antibody 12CA5 (1:2000). Images were acquired with ChemiDoc (Bio-Rad) and analyzed using Image Lab.

### Drug sensitivity assay

Exponentially growing cultures were serially diluted 10-fold in water, and 5 μl of each dilution was spotted onto YEPD plates with or without DNA-damaging agents. Images were acquired after 3 days of incubation at 25°C. Each experiment was performed at least twice.

### Structural bioinformatics

Tel1 structures were retrieved from the Protein Data Bank (PDB IDs: 6S8F, 6JXA, and 6JXC). A gap-free model of the Tel1 dimer was reconstructed using the AlphaFold 3 platform [34] and used to generate images with UCSF Chimera software [35]. A complete model of the FAT and KIN domains of an asymmetric Tel1 protomer was built using the homology-modeling pipeline implemented in the Maestro suite (Schrödinger, LLC, New York, NY, 2025), with the available Tel1 structure as a template [36, 37]. A truncated structural model of Tel1-400 spanning residues 1601–2787 was generated by introducing the point mutations into the wild-type Tel1 structure (PDB ID: 6S8F) with UCSF Chimera, followed by structural minimization with MacroModel (Schrödinger Release 2026-2: MacroModel, Schrödinger, LLC, New York, NY, 2025). Minimizations were carried out using the AMBER force field [38] with implicit solvent, the PRCG method, a maximum of 2500 iterations, and a gradient convergence threshold of 0.05. Canonical coarse-grained molecular dynamics (CG-MD) simulations were performed using the UNRES model on the UNRES server, version 23.01.2025 (https://unres-server.chem.ug.edu.pl) [39]. CG-MD simulations were performed with the NEWCT-9P UNRES force field, using a simulation step of 48.9 fs, at three different temperatures (288, 290, and 295 K). Simulations were initiated either from the model generated with AlphaFold 3 for wild-type Tel1 or from the Tel1-400 model prepared as described above, both spanning residues 1601–2787, and were run for at least 10 ns each. The root mean square deviation (RMSD) profiles showed that a plateau was reached within 6 ns in all simulations. The radius of gyration also showed a rapid change within the first 4 ns and remained more stable thereafter (Supplementary Fig. S1). Residual variations in RMSD or radius of gyration were associated with movements of hyperflexible substructures, such as the LID coiled-coils. Trajectory movies were generated after aligning the Cα atoms of each 0.0489-ns frame to a reference structure to remove translational and rotational motion. A five-frame moving average of atomic coordinates was applied to reduce high-frequency fluctuations in the trajectories using PyMOL software (The PyMOL Molecular Graphics System, Version 3.0 Schrödinger, LLC). All trajectory analyses were performed using dedicated tools in the UCSF Chimera suite.

### Kinase assay

Kinase assays were performed as previously described [40]. Protein extracts were incubated with 50 μl of Dynabeads Protein G (Thermo Fisher Scientific) for 2 h at 4°C in the presence of 5 μg of anti-HA (12CA5) antibodies. Kinase reactions were incubated at 30°C for 30 min. Sodium dodecyl sulfate (SDS) gel-loading buffer was added to the samples, and bound proteins were resolved by electrophoresis on an 18% SDS–polyacrylamide gel and visualized by autoradiography. The remaining 300 μl of each bead suspension was collected, dried, resuspended in 10 μl of loading buffer, boiled, and analyzed by western blotting with anti-HA antibodies.

### Chromatin immunoprecipitation and qPCR

YEPR exponentially growing cell cultures of JKM139 derivative strains, carrying an HO cleavage site at the *MAT* locus, were transferred to YEPRG at time zero. Crosslinking was done with 1% formaldehyde for 10 min (Rad9, Rad9-7xA, and Rad50) or 15 min (Tel1, Tel1-400, and Sgs1). The reaction was stopped by adding 0.125 M glycine for 5 min. Immunoprecipitation was performed by incubating samples with Dynabeads Protein G (Thermo Fisher Scientific) for 3 h at 4°C in the presence of 5 μg of anti-HA (12CA5) antibodies. Immunoprecipitated DNA was quantified by qPCR using a Bio-Rad CFX Connect^™^ Real-Time PCR Detection System and CFX Maestro 1.1 software. qPCR reactions were performed in triplicate in a final volume of 20 μl containing 10 ng of template DNA, 300 nM of each primer, and 2X SsoFast^™^ EvaGreen Supermix (Bio-Rad #1725201; 2X reaction buffer containing dNTPs, Sso7d-fusion polymerase, MgCl_2_, EvaGreen dye, and stabilizers). Reactions were run in white 96-well Multiplate^™^ PCR plates (Bio-Rad #MLL9651). The qPCR program was as follows: step 1, 98°C for 2 min; step 2, 90°C for 5 s; step 3, 60°C for 15 s; step 4, return to step 2 and repeat 40 times. At the end of the cycling program, a melting-curve analysis (from 65°C to 95°C with a 0.5°C increment every 5 s) was run to test the specificity of each qPCR. Data are expressed as fold enrichment at the HO-induced DSB relative to the uncleaved *ARO1* locus, after normalization of each chromatin immunoprecipitation (ChIP) signal to the corresponding input. Fold-enrichment values were subsequently normalized to DSB induction efficiency at each time point. Oligonucleotides used for qPCR analyses are listed in Supplementary Table S3.

### FKBP dimerization

To induce FKBP-mediated dimerization, exponentially growing cells were treated with 1 μM AP20187 (ARGENT Regulated Homodimerization Kit, ARIAD Pharmaceuticals). For spot assays, cells were treated with AP20187 for 6 h and then spotted onto YEPD plates containing or lacking camptothecin (CPT). For DSB end-tethering assays, cells were treated with 1 μM AP20187 for 6 h and then arrested in G1 with α-factor or in G2 with nocodazole. DSB end-tethering was analyzed after 0, 1, and 2 h, with cells maintained in the presence of AP20187 together with α-factor or nocodazole, respectively. For chromatin immunoprecipitation experiments, AP20187-treated cells were transferred to YEPRG medium to induce HO expression, and samples were collected at the indicated time points for chromatin immunoprecipitation followed by qPCR. AP20187 was present throughout the experiment.

### DSB repair by single-strand annealing and ectopic recombination

DSB repair by SSA was detected in YMV45 derivative strains by Southern blot analysis using an Asp718-SalI fragment containing part of the *LEU2* gene as a probe. DSB repair was quantified as the ratio of the SSA product to the sum of the SSA and DSB products at each time point. To correct for cleavage efficiency, the uncut-band signal was subtracted from the total signal before calculating the fraction of SSA product. DSB repair by ectopic recombination was detected by using the tGI354 strain. To determine repair efficiency, the intensities of the NCO and CO bands at each time point were normalized to a loading control and corrected for the amount of uncut substrate remaining at 2 h after HO induction, when DSB formation was maximal. The resulting values were expressed relative to the normalized intensity of the uncut *MAT*a band at time zero, which was set to 100%. Densitometric analysis of band intensities was performed using Scion Image Beta 4.0.2 software.

### DSB resection

YEPR exponentially growing cell cultures of JKM139 derivative strains, carrying the HO-cut site at the *MAT* locus, were transferred to YEPRG at time zero. SspI-digested genomic DNA was run on an alkaline agarose gel and visualized after hybridization with an RNA probe that anneals with the unresected strand on one side of the HO-induced DSB as previously described [41]. This probe was obtained by *in vitro* transcription using Promega Riboprobe System-T7 and plasmid pML514 as a template. Plasmid pML514 was constructed by inserting a 900-bp fragment containing part of the *MAT* locus (coordinates 200 870 to 201 587 on chromosome III) into the pGEM7Zf vector. Quantitative analysis of DSB resection was performed by calculating the ratio of the ssDNA-band intensities to the total intensity of DSB-derived products. The resection efficiency was normalized with respect to the HO cleavage efficiency for each time point. Densitometric analysis of band intensities was performed using Scion Image Beta 4.0.2 software.

### Statistical analysis

Statistical analysis was performed using Microsoft Excel Professional 365 software. *P*-values were determined using an unpaired, two-tailed Student’s *t*-test. No statistical methods were used to predetermine sample size, and no samples were excluded from the analyses.

## Results

### The *tel1-400* allele suppresses the DNA damage sensitivity of *sae2*Δ cells and increases Tel1 association with DSBs

Cells lacking Sae2 are defective in DSB end processing and display persistent DSB-induced checkpoint activation [22, 24, 31]. This heightened checkpoint signaling stems from sustained MRX and Tel1 association with DNA breaks, which promotes Rad9 accumulation at DSBs and prolonged Rad53 phosphorylation [22, 24]. We previously showed that loss of Tel1 kinase activity suppresses the DNA damage sensitivity of *sae2*Δ cells by reducing Rad9 occupancy at DSBs [28]. Reduced Rad9 accumulation dampens Rad53 signaling and promotes re-entry into the cell cycle [21, 28]. Furthermore, because Rad9 limits Sgs1 association with DSBs, reduced Rad9 occupancy relieves this inhibition, allowing Sgs1–Dna2 to partially compensate for the resection defect of *sae2*Δ cells [20, 21, 29].

**Figure 1.**
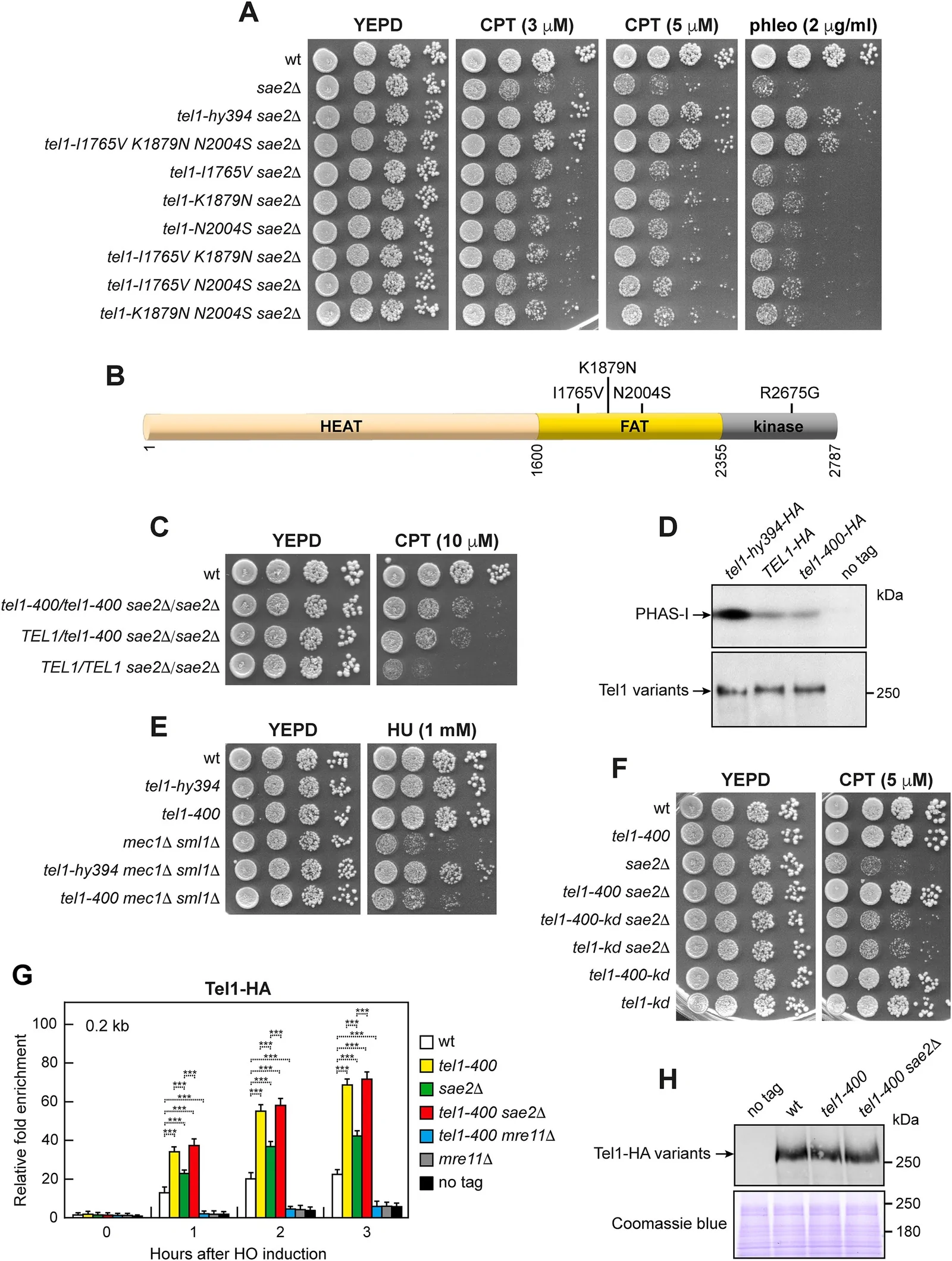
*tel1-400* reduces the DNA damage sensitivity of *sae2*Δ cells and increases Tel1 association with DSBs. (**A**) Drug-sensitivity assay. Exponentially growing cultures were serially diluted (1:10) and spotted onto yeast extract, bactopeptone, and glucose (YEPD) plates with or without CPT or phleomycin. (**B**) Schematic representation of Tel1 domains, indicating the positions of the *tel1-hy394* mutations. (**C**) Drug-sensitivity assay. Exponentially growing cultures were serially diluted (1:10) and spotted onto YEPD plates with or without CPT. (**D**) *In vitro* kinase assay. Tel1–HA variants were immunoprecipitated with an anti-HA antibody and incubated with PHAS-I and γ-^32^P-labeled ATP, followed by SDS–PAGE. Immunoprecipitates were also analyzed by western blot with an anti-HA antibody. (**E** and **F**) Drug-sensitivity assay. Exponentially growing cultures were serially diluted (1:10) and spotted onto YEPD plates with or without HU or CPT. (**G**) ChIP-qPCR analysis of Tel1 occupancy at an HO-induced DSB. YEPR cultures of JKM139-derived strains were transferred to YEPRG to induce HO expression. Relative Tel1–HA enrichment at the indicated distance from the HO cut site was measured after anti-HA ChIP followed by qPCR. Bars represent the mean ± s.d. from three independent experiments. ***P < 0.005 (unpaired two-tailed Student’s *t*-test). (**H**) Western blot analysis of Tel1–HA variants in extracts used for the ChIP in panel (G), probed with an anti-HA antibody. Coomassie Blue staining of the corresponding SDS–PAGE gel is shown as a loading control.

To further investigate the contribution of Tel1 to the DNA damage sensitivity of *sae2*Δ cells, we tested a set of hypermorphic *tel1* alleles, referred to as *tel1-hy*, previously identified by their ability to partially suppress the hydroxyurea (HU) sensitivity and checkpoint defects of *mec1*Δ cells kept viable by *SML1* deletion [40]. Among these, *tel1-hy394* suppressed *sae2*Δ genotoxin sensitivity, as *tel1-hy394 sae2*Δ cells were more resistant than *sae2*Δ cells to CPT, which traps Top1 cleavage complexes and generates replication-dependent DNA breaks, and to phleomycin, a radiomimetic drug that directly induces DNA strand breaks (Fig. 1A).

Tel1/ATM belongs to the phosphatidylinositol 3-kinase-like kinase (PIKK) family, whose members contain a two-lobed kinase domain with conserved regulatory elements [11]. N-terminal to the kinase domain, Tel1 contains the FAT (FRAP, ATM, TRRAP) domain, whereas the remaining regions are largely composed of α-helical HEAT repeats (Fig. 1B). The *tel1-hy394* allele carries three substitutions in the FAT domain (I1765V, K1879N, and N2004S) and one substitution in the kinase domain (R2675G) (Fig. 1B).

To determine which change(s) were responsible for suppression, we first separated the FAT- and kinase-domain mutations. Cells carrying only the three FAT-domain substitutions suppressed the DNA damage sensitivity of *sae2*Δ cells to an extent similar to that of the original *tel1-hy394* allele, indicating that the R2675G substitution is dispensable for suppression (Fig. 1A). We then dissected the contribution of the individual FAT-domain mutations by testing each substitution alone and in all pairwise combinations in the *sae2*Δ background. No individual substitution or pairwise combination suppressed *sae2*Δ genotoxin sensitivity (Fig. 1A). Instead, suppression required the simultaneous presence of all three FAT-domain substitutions, I1765V, K1879N, and N2004S, hereafter referred to as *tel1-400* (Fig. 1A).

*tel1-400*-mediated suppression was dominant, as *TEL1*/*tel1-400 sae2*Δ/*sae2*Δ diploid cells were less sensitive to CPT than *TEL1*/*TEL1 sae2*Δ/*sae2*Δ diploid cells (Fig. 1C).

Because the *tel1-hy394* allele was reported to increase intrinsic Tel1 kinase activity [40], we compared the *in vitro* kinase activities of immunoprecipitated Tel1–HA variants using PHAS-I as a substrate [42]. As expected, Tel1-hy394 displayed increased kinase activity, whereas Tel1-400 showed activity comparable to that of wild-type Tel1 when equivalent amounts of protein were present in the immunoprecipitates (Fig. 1D). Thus, suppression of *sae2*Δ genotoxin sensitivity by *tel1-400* does not require the elevated Tel1 kinase activity likely conferred by the R2675G mutation. Consistently, *tel1-hy394*, but not *tel1-400*, suppressed the HU sensitivity of *mec1*Δ *sml1*Δ cells (Fig. 1E), indicating that Mec1 bypass requires the elevated Tel1 activity associated with Tel1-hy394.

Suppression nonetheless required Tel1 catalytic function. Introducing kinase-dead substitutions (G2611D, D2612A, N2616K, and D2631E), which abolish Tel1 activity *in vitro* [42], into *tel1-400* generated a *tel1-400-kd* allele that no longer suppressed *sae2*Δ DNA damage sensitivity. Indeed, *tel1-400-kd sae2*Δ cells were more CPT-sensitive than *tel1-400 sae2*Δ cells and as sensitive as *tel1-kd sae2*Δ cells (Fig. 1F). Together, these results indicate that *tel1-400*-mediated suppression of *sae2*Δ requires Tel1 catalytic activity but not an increase in intrinsic kinase activity above wild-type levels.

Finally, we tested whether *tel1-400* affects Tel1 association with DSBs by introducing the allele into the JKM139 background, in which galactose-induced HO expression generates a single irreparable DSB at the *MAT* locus because the homologous donor loci *HML* and *HMR* are deleted. ChIP followed by quantitative PCR (qPCR) showed that, as expected, *SAE2* deletion increased Tel1 association with the HO-induced DSB (Fig. 1G). The *tel1-400* allele resulted in a more pronounced increase in Tel1 occupancy than *SAE2* deletion, with comparable enrichment observed in *tel1-400* and *tel1-400 sae2*Δ cells (Fig. 1G). This increased occupancy was not due to elevated Tel1 abundance, as Tel1 protein levels were comparable in wild-type, *tel1-400*, and *tel1-400 sae2*Δ cells (Fig. 1H), indicating that the FAT-domain substitutions enhance Tel1 association with DNA damage sites. Tel1-400 enrichment at the DSB still required the MRX complex, as does wild-type Tel1 [43], because deletion of *MRE11* abolished the association of both Tel1 and Tel1-400 with the HO-induced DSB (Fig. 1G).

### Structural mapping and dynamic effects of Tel1-400 substitutions

**Figure 2.**
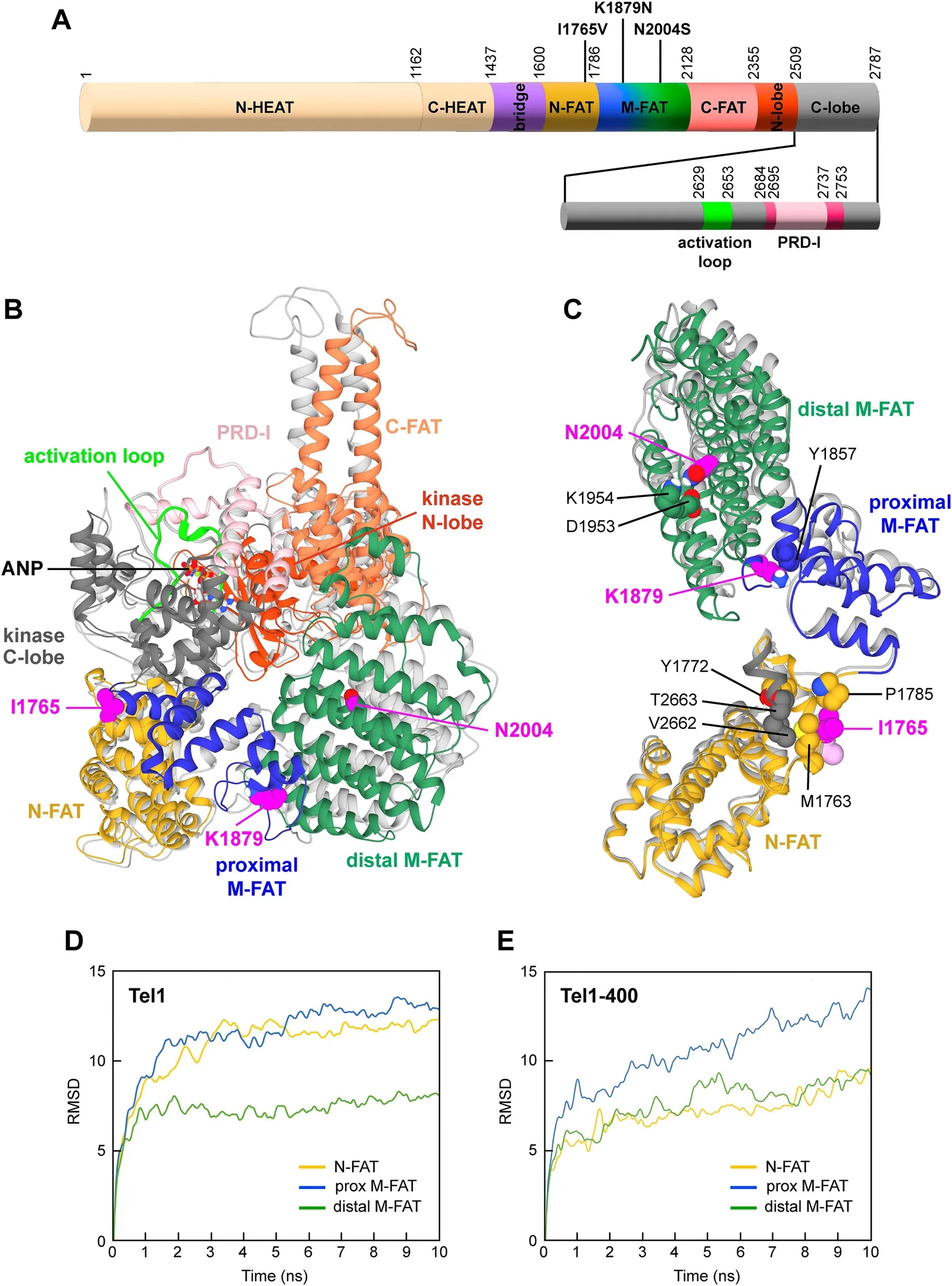
Tel1-400 substitutions map to distinct regions within the FAT domain. (**A**) Schematic representation of Tel1 domains. The inset enlarges the kinase C-lobe and highlights conserved PIKK regulatory elements, including the activation loop (bright green) and the PRD (magenta), with its inhibitory PRD-I loop shown in pink. (**B**) Superposition of two Tel1 conformations aligned on the kinase-domain C-lobe: a subunit from the inactive dimeric Tel1 structure (colored; PDB ID: 6S8F) and an active-like asymmetric Tel1 conformation (light gray; complete model based on PDB ID: 6JXA). The FAT domain is divided into three subregions: N-FAT (residues 1601–1786; yellow ochre); M-FAT, which is subdivided into a proximal segment (residues 1787–1870; blue) and a distal segment (residues 1871–2128; green); and C-FAT (residues 2129–2355; coral), comprising the LID and the HRD. The kinase domain comprises the N-lobe (residues 2356–2509; reddish orange) and the C-lobe (residues 2510–2787; gray). The PRD-I loop (residues 2696–2737; pink) is fully disordered in the active-like asymmetric Tel1 structure [37]. The ATP analog ANP (adenosine 5′-[β,γ-imido]triphosphate) is shown as sticks, and the activation loop is shown in bright green. Mutated residues are shown as magenta spheres. (**C**) Zoomed view of the Tel1 N-FAT and M-FAT regions (residues 1601–2128), showing the positions of the Tel1-400 substitutions. Mutated residues are shown as magenta spheres, and selected surrounding residues are shown as spheres. The portion of the kinase C-lobe that contacts N-FAT is shown in gray. The superimposed active-like asymmetric Tel1 structure is shown as a semi-transparent light-gray cartoon, except for I1765, which is shown as pink spheres. In panels (B) and (C), oxygen is shown in red, nitrogen in blue, hydrogen in white, phosphorus in orange, and Mg^2+^ as a green sphere. (**D**) RMSD profiles of the indicated subregions of wild-type Tel1 (residues 1601–2787) along the 290 K CG-MD trajectory. (**E**) RMSD profiles of the indicated subregions of Tel1-400 (residues 1601–2787) along the 290 K CG-MD trajectory.

The Tel1 HEAT repeats can be divided into N-HEAT and C-HEAT bodies, which are connected through a bridge region to the FAT and kinase domains (Fig. 2A). The FAT domain can be subdivided into N-FAT, M-FAT, and C-FAT, whereas the kinase domain comprises an N-lobe and a C-lobe. The C-lobe contains conserved regulatory elements, including the activation loop and the PIKK regulatory domain (PRD), whose inhibitory segment, the PRD-I loop, can occlude substrate access to the active site (Fig. 2A) [44].

The three residues substituted in Tel1-400 map within the FAT domain: I1765 lies in N-FAT at the interface with the kinase C-lobe, whereas K1879 and N2004 map within M-FAT (Fig. 2B and C). Direct DNA binding has been reported for Tel1 and other PIKK-family members [45]. However, the DNA-contacting surfaces identified so far are generally located within N-terminal regions rather than within the FAT domain [46, 47], arguing against the possibility that Tel1-400 acts simply by increasing intrinsic DNA binding.

In the most recent inactive Tel1 structure (Supplementary Fig. S2A and B), I1765 lies at the N-FAT-kinase interface and makes hydrophobic contacts with residues in both N-FAT, including M1763, Y1772, and P1785, and the kinase C-lobe, including V2662 and T2663 (Fig. 2C) [36]. Comparison of the inactive and active-like asymmetric *S. cerevisiae* Tel1 conformations (Supplementary Fig. S2A and B for the inactive conformation, C and D for the active-like conformation) indicates that these contacts are disrupted during kinase-domain opening (see detail in Fig. 2C) [37]. In the active-like conformation, compression of the N-terminal HEAT domain and FAT-domain repositioning move I1765 away from the kinase C-lobe, while the inhibitory PRD-I segment becomes disordered and the kinase domain adopts a more open, substrate-accessible arrangement [37]. This rearrangement is accompanied by repositioning of the kinase N-lobe relative to the C-lobe and tilting of the LBE-interacting domain (LID), a C-FAT element that contacts the LBE of the kinase N-lobe, toward the C-lobe (Fig. 2B). A related open kinase configuration was recently observed in one subunit of a dimeric *S. pombe* Tel1 complex bound to a CHK2 substrate peptide [48]. Thus, I1765 lies at a dynamic FAT-kinase interface that is remodeled during the transition toward an open kinase state, suggesting that the I1765V substitution may alter local packing and/or mechanical coupling between N-FAT and the kinase domain.

The other two substitutions map within M-FAT. K1879 lies close to Y1857, raising the possibility that K1879N affects local interactions within this region, whereas N2004 maps to a more distal M-FAT segment positioned between the FAT scaffold and the kinase module (Fig. 2C). Although their precise structural consequences remain unclear, these locations suggest that *tel1-400* may affect multiple sites within the FAT-kinase coupling region.

To investigate whether the Tel1-400 substitutions alter FAT-domain dynamics, CG-MD simulations were performed on wild-type Tel1 and Tel1-400 models spanning residues 1601–2787. Simulations were run for 10 ns at 288, 290, and 295 K. Since the trajectories obtained at different temperatures showed similar overall motions, only the 290 K simulation trajectories are shown in Supplementary Movies S1 and S2.

In wild-type Tel1, root mean square deviation (RMSD) analysis showed that N-FAT, proximal M-FAT, and distal M-FAT rapidly underwent a coordinated rearrangement (Fig. 2D and Supplementary Movie S1). N-FAT and proximal M-FAT showed similar RMSD kinetics and reached comparable plateau values, whereas distal M-FAT showed a more limited displacement. Inspection of the trajectory indicated that N-FAT rotated toward the kinase domain, proximal M-FAT rearranged around K1879, distal M-FAT accommodated N-FAT movement by rotating on itself, and the LID tilted toward the kinase C-lobe.

In the Tel1-400 model, the FAT subregions displayed broadly similar motions but with altered relative kinetics (Fig. 2E and Supplementary Movie S2). N-FAT no longer moved in concert with the M-FAT subregions, whereas proximal M-FAT showed an earlier and larger displacement, consistent with altered local anchoring around K1879. Moreover, whereas distal M-FAT behaved as a relatively coordinated unit in wild-type Tel1, it separated into two kinetically distinct regions in Tel1-400: distal M-FAT I (residues 1880–1986) initially showed lower displacement and displayed kinetics more similar to those of N-FAT, whereas distal M-FAT II (residues 1987–2128) moved rapidly and followed a distinct trajectory (Supplementary Fig. S3A and B). This altered behavior may reflect weakened interactions within M-FAT, possibly involving loss of K1879-mediated anchoring and/or changes in contacts involving N2004 and nearby residues.

Together, these structural and dynamic analyses suggest that the *tel1-400* substitutions do not grossly disrupt Tel1 structure or perturb a single local contact. Instead, they are associated with altered coordination of FAT-subregion motions and changes in the dynamic coupling between the FAT scaffold and the kinase domain, consistent with the Tel1-400 substitutions altering Tel1 conformational dynamics.

### The *tel1-400* allele neither suppresses checkpoint persistence nor restores DSB resection in *sae2*Δ cells

Cells lacking Sae2 display persistent DSB-induced checkpoint activation due to increased MRX and Tel1 association with DNA breaks, which promotes Rad9 accumulation and sustained Rad53 phosphorylation, ultimately leading to permanent cell-cycle arrest [22, 24]. Because checkpoint recovery can be influenced by DSB repair efficiency, we asked whether *tel1-400* affects checkpoint regulation independently of repair proficiency. To uncouple checkpoint signaling from DNA repair, we introduced *tel1-400* into the JKM139 background, in which galactose-induced HO expression generates a single irreparable DSB at the *MAT* locus because the donor loci *HML* and *HMR* are deleted. In this system, checkpoint extinction can occur only through adaptation, defined as re-entry into the cell cycle and decline in Rad53 phosphorylation despite persistence of the unrepaired DSB [49–51].

**Figure 3.**
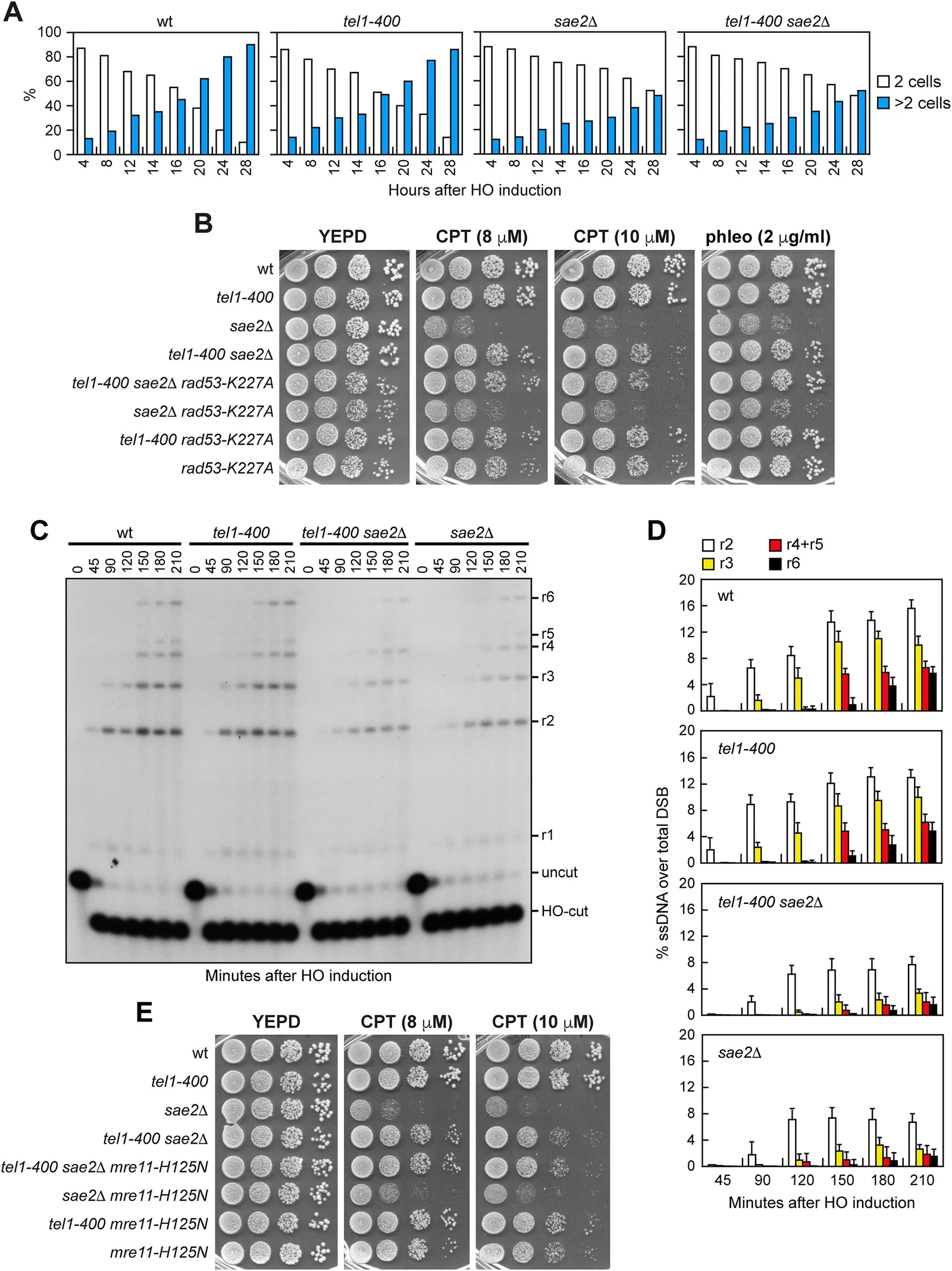
*tel1-400* neither suppresses checkpoint persistence nor restores DSB resection in *sae2*Δ cells. (**A**) Adaptation assay. G1-arrested yeast extract-peptone-raffinose (YEPR) cultures of JKM139 derivative strains were plated onto galactose-containing plates (time zero). At the indicated times, 200 cells per strain were scored for two-cell dumbbells (2 cells) and microcolonies (>2 cells). (**B**) Drug-sensitivity assay. Exponentially growing cultures were serially diluted (1:10) and spotted onto YEPD plates with or without CPT or phleomycin. (**C**) DSB resection assay. Exponentially growing YEPR cultures of JKM139 derivative strains were transferred to yeast extract, peptone, raffinose, and galactose (YEPRG) at time zero to induce HO. SspI-digested genomic DNA was separated on an alkaline agarose gel and hybridized with a strand-specific *MAT* probe complementary to the unresected strand. Progressive 5′–3′ resection eliminates SspI sites, generating longer SspI fragments (r1–r6) detected by the probe. (**D**) Densitometric analysis of the resection products shown in panel (C). Bars represent the mean ± s.d. from three independent experiments. (**E**) Drug-sensitivity assay. Exponentially growing cultures were serially diluted (1:10) and spotted onto YEPD plates with or without CPT.

G1-arrested cells were plated on galactose-containing medium to induce HO. Within 4 h, most wild-type, *tel1-400, sae2*Δ, and *tel1-400 sae2*Δ cells accumulated as large-budded cells (Fig. 3A), indicating checkpoint activation in all strains. By 24 h, wild-type and *tel1-400* cells adapted and formed microcolonies containing more than two cells, whereas comparable fractions of *sae2*Δ and *tel1-400 sae2*Δ cells remained arrested as two-cell dumbbells (Fig. 3A), in agreement with previous evidence that elevated Rad53 signaling prevents adaptation in *sae2*Δ cells [22, 24]. Accordingly, Rad53 phosphorylation, detected as reduced electrophoretic mobility, persisted for at least 24 h after HO induction in both *sae2*Δ and *tel1-400 sae2*Δ cells (Supplementary Fig. S4). Thus, *tel1-400* does not alleviate the persistent checkpoint response of *sae2*Δ cells under conditions in which DSB repair cannot occur.

Consistent with this conclusion, *tel1-400* still suppressed the DNA damage sensitivity of *sae2*Δ cells carrying the kinase-defective *rad53-K227A* allele (Fig. 3B), indicating that *tel1-400*-mediated suppression does not require Rad53 activity.

We next asked whether *tel1-400* suppresses *sae2*Δ DNA damage sensitivity by restoring DSB resection. Resection at the HO-induced break was monitored by the accumulation of SspI-resistant ssDNA fragments, r1–r6, detected by alkaline gel electrophoresis and Southern blotting using a probe complementary to the unresected strand on one side of the DSB (Supplementary Fig. S5). As expected, *sae2*Δ cells showed reduced accumulation of ssDNA intermediates relative to wild-type, and this defect was not rescued by *tel1-400*, as *tel1-400 sae2*Δ cells exhibited similarly impaired ssDNA formation (Fig. 3C and D). Therefore, *tel1-400* does not suppress *sae2*Δ by restoring DSB resection.

Finally, because Sae2 stimulates Mre11 endonuclease-dependent processing at DNA ends [9], we tested whether *tel1-400* bypasses Sae2 by restoring Mre11 nuclease function. *tel1-400* still suppressed the DNA damage sensitivity of *sae2*Δ cells carrying the nuclease-defective *mre11-H125N* allele (Fig. 3E), indicating that suppression does not result from restored Mre11 nuclease-dependent DNA-end processing.

### The *tel1-400* allele suppresses the DSB end-tethering defect of *sae2*Δ cells in a Rad9-dependent manner and enhances DNA damage-induced Rad9 phosphorylation

In addition to promoting DSB resection, Sae2 is required to maintain broken DNA ends in close proximity, an activity that supports accurate repair by both HR and NHEJ [31–33]. We therefore asked whether *tel1-400* suppresses the end-tethering defect of *sae2*Δ cells. To visualize genomic regions flanking a DSB, we used strains constitutively expressing LacI-GFP and carrying LacO arrays integrated 50 kb upstream and downstream of an irreparable HO cleavage site [52]. DSB end-tethering was scored microscopically: a single LacI-GFP focus indicates juxtaposed and tethered DNA ends, whereas two foci indicate separated, untethered ends. To exclude defects in sister-chromatid cohesion as a cause of two foci, HO expression was induced by galactose addition in cultures arrested in G1 with α-factor and maintained in G1 throughout the experiment. We also performed the assay in nocodazole-arrested cells to determine whether *tel1-400* could restore DSB end-tethering in G2, when DSB end resection can occur.

**Figure 4.**
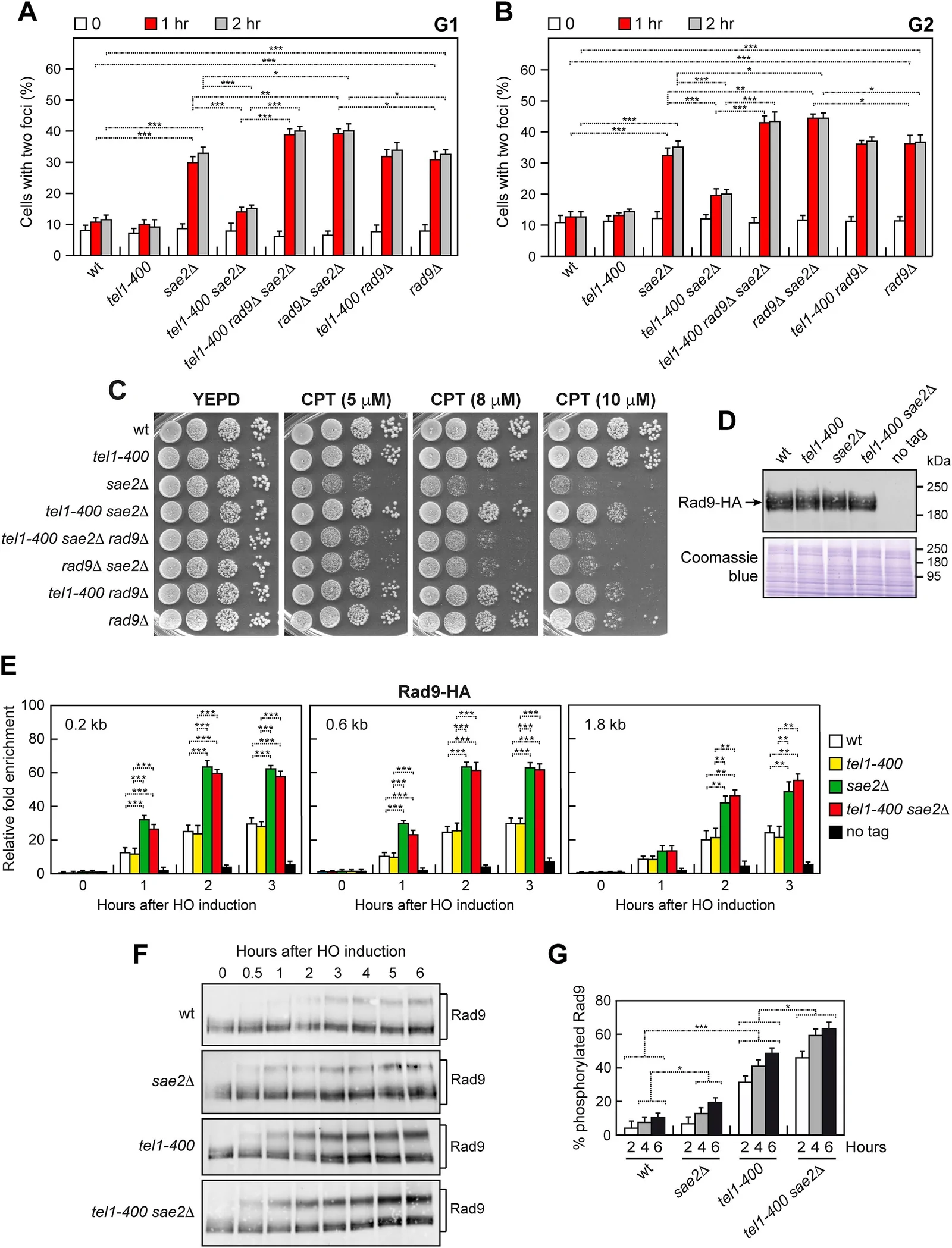
*tel1-400* suppresses the end-tethering defect of *sae2*Δ cells through Rad9. (**A** and **B**) DSB end-tethering assay. Exponentially growing YEPR cultures were arrested in G1 with α-factor (time zero) (A) or in G2 with nocodazole (B), and shifted to YEPRG in the continuous presence of α-factor or nocodazole, respectively. For each strain, 200 cells were scored for the percentage of cells displaying two LacI-GFP foci. Bars represent the mean ± s.d. from three independent experiments; ***P < 0.005, **P < 0.01, *P < 0.05 (unpaired two-tailed Student’s *t*-test). (**C**) Drug-sensitivity assay. Exponentially growing cultures were serially diluted (1:10) and spotted onto YEPD plates with or without CPT. (**D**) Western blot analysis of Rad9–HA probed with an anti-HA antibody. Coomassie Blue staining of the corresponding SDS–PAGE gel is shown as a loading control. (**E**) ChIP-qPCR analysis of Rad9 occupancy at an HO-induced DSB. Relative Rad9–HA enrichment at the indicated distance from the HO cut site was measured after anti-HA ChIP followed by qPCR. Bars represent the mean ± s.d. from three independent experiments; ***P < 0.005, **P < 0.01 (unpaired two-tailed Student’s *t*-test). (**F**) Rad9 phosphorylation after induction of an irreparable HO-induced DSB. HO expression was induced by galactose addition, and protein extracts were analyzed by western blot using an anti-HA antibody. (**G**) Densitometric analysis of Rad9 phosphorylation shown in panel (F). Densitometry was performed by calculating the ratio of band intensities for slowly migrating bands to the total Rad9 signal. Bars represent the mean ± s.d. from three independent experiments; ***P < 0.005, *P < 0.05 (unpaired two-tailed Student’s *t*-test).

As previously reported [31, 33], G1-arrested *sae2*Δ cells showed an increased fraction of cells with two LacI-GFP foci after HO induction, whereas wild-type cells predominantly displayed a single focus (Fig. 4A). Introduction of *tel1-400* into the *sae2*Δ background significantly reduced the fraction of cells with two foci, indicating that *tel1-400* suppresses the *sae2*Δ end-tethering defect in G1-arrested cultures (Fig. 4A). Similar results were obtained in nocodazole-arrested G2 cells, in which *sae2*Δ cells showed increased DSB end separation after HO induction, and this defect was significantly reduced by *tel1-400* (Fig. 4B).

This suppression required Rad9, as *tel1-400* failed to reduce end separation in both G1- and G2-arrested *rad9*Δ *sae2*Δ cells (Fig. 4A and B). Consistent with this genetic interaction, Rad9 was also necessary for *tel1-400*-mediated suppression of genotoxin sensitivity, since *tel1-400 rad9*Δ *sae2*Δ cells displayed DNA damage sensitivity comparable to that of *rad9*Δ *sae2*Δ cells (Fig. 4C). Notably, *RAD9* deletion increased the fraction of cells with separated DSB ends after HO induction in both G1- and G2-arrested wild-type and *tel1-400* cells, and further increased end separation in *sae2*Δ cells (Fig. 4A and B), supporting a role for Rad9 in DSB end-tethering that is distinct from that of Sae2.

Next, we asked whether *tel1-400* alters Rad9 abundance or recruitment to DSBs. *tel1-400* did not affect Rad9 abundance in either the *SAE2* or *sae2*Δ background, as comparable Rad9 levels were detected in protein extracts from wild-type and mutant cells (Fig. 4D). ChIP-qPCR analysis showed that Rad9 occupancy at the HO-induced DSB was similar in wild-type and *tel1-400* cells (Fig. 4E). As expected, Rad9 association with DSBs increased in *sae2*Δ cells, and *tel1-400* did not further enhance Rad9 occupancy in the *sae2*Δ background (Fig. 4E), indicating that *tel1-400* does not act by enhancing Rad9 loading at DNA breaks.

DNA damage induces checkpoint-mediated Rad9 phosphorylation on multiple [S/T]Q sites within the SCD, including T603, and these phosphorylation events promote BRCT–SCD interactions and Rad9 oligomerization [19]. Rad9 phosphorylation, detected as a mobility shift after HO induction, was slightly enhanced in *sae2*Δ cells compared to wild-type cells. This phosphorylation was further increased in *tel1-400* cells and was highest in *tel1-400 sae2*Δ cells (Fig. 4F and G), indicating that *tel1-400* enhances and/or prolongs DNA damage-induced Rad9 phosphorylation.

### Rad9 oligomerization is required for DSB end-tethering

Because Rad9 phosphorylation, which promotes oligomerization, is increased in *tel1-400* cells, we asked whether Rad9 supports DSB end-tethering through its ability to self-associate at sites of damage. To test this hypothesis, we first analyzed the *rad9-7xA* allele, in which six SQ/TQ sites in the SCD (T390, T398, T410, T427, S435, and T457), together with T603, are mutated to alanine [16]. This mutant prevents phosphorylation at these key Mec1/Tel1 target sites and impairs Rad9 oligomerization by disrupting BRCT–SCD interactions [16, 19, 29].

**Figure 5.**
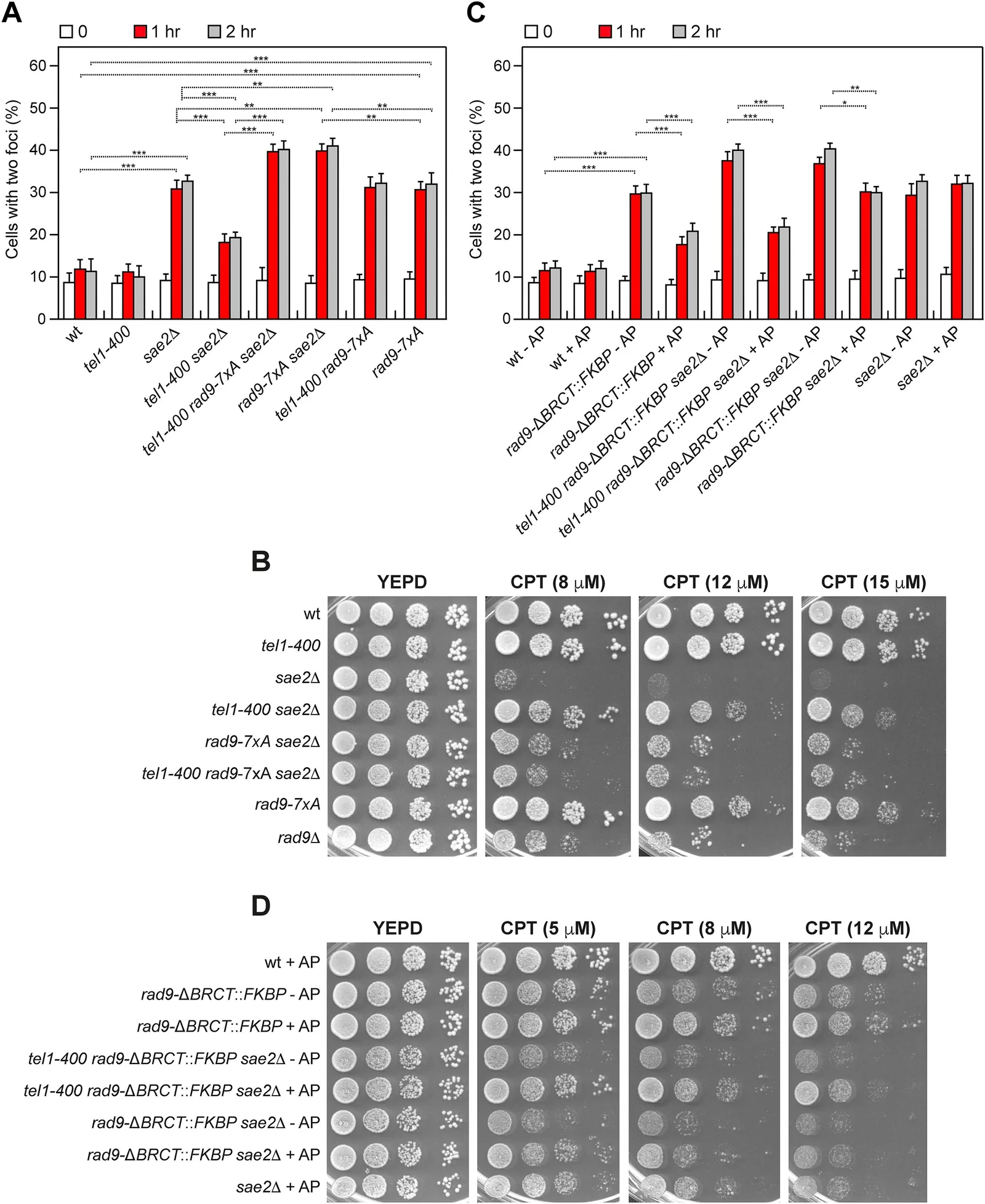
Rad9 oligomerization is required for *tel1-400*-dependent restoration of end-tethering and damage resistance. (**A**) DSB end-tethering assay. Exponentially growing YEPR cultures were arrested in G1 with α-factor (time zero) and shifted to YEPRG in the continuous presence of α-factor. For each strain, 200 cells were scored for the percentage of cells displaying two LacI-GFP foci. Bars represent the mean ± s.d. from three independent experiments; ***P < 0.005, **P < 0.01 (unpaired two-tailed Student’s *t*-test). (**B**) Drug-sensitivity assay. Exponentially growing cultures were serially diluted (1:10) and spotted onto YEPD plates with or without CPT. (**C**) DSB end-tethering assay performed as in panel (A). Bars represent the mean ± s.d. from three independent experiments; ***P < 0.005, **P < 0.01, *P < 0.05 (unpaired two-tailed Student’s *t*-test). (**D**) Drug-sensitivity assay. Exponentially growing cultures were left untreated or treated with AP20187 for 6 h, serially diluted (1:10), and spotted onto YEPD plates with or without CPT.

When HO was induced in G1-arrested cultures that were maintained in G1 throughout the experiment, *rad9-7xA* cells showed an increased fraction of cells with two LacI-GFP foci after DSB induction, and this defect was further exacerbated by *SAE2* deletion (Fig. 5A). Similar results were obtained in G2-arrested cells (Supplementary Fig. S6). Thus, impairment of Rad9 SCD phosphorylation causes a DSB end-tethering defect in otherwise wild-type cells and further exacerbates end separation in *sae2*Δ cells, indicating that Rad9 phosphorylation-dependent oligomerization contributes to end-tethering and helps maintain residual DSB-end proximity when Sae2-dependent tethering is compromised.

This defect is unlikely to result from impaired Rad9 association with DSBs or altered bulk resection. Indeed, the *rad9-7xA* mutations did not alter Rad9 protein levels (Supplementary Fig. S7A), and Rad9-7xA showed DSB recruitment kinetics and levels comparable to those of wild-type Rad9 (Supplementary Fig. S7B). Moreover, wild-type and *rad9-7xA* cells showed similar DSB resection kinetics (Supplementary Fig. S7C). Consistent with the oligomerization defect of Rad9-7xA, *rad9-7xA* cells showed defective checkpoint activation after DSB induction (Supplementary Fig. S7D), in agreement with previous data showing that Rad9 assembly into higher-order oligomers promotes stable Rad9–Rad53 association and Rad53 autophosphorylation [12–19].

Importantly, *tel1-400* failed to reduce DSB end separation in *rad9-7xA* or *rad9-7xA sae2*Δ cells arrested in either G1 (Fig. 5A) or G2 (Supplementary Fig. S6), indicating that *tel1-400*-mediated restoration of end-tethering requires the Rad9 SCD phosphorylation sites that support Rad9 oligomerization. Consistent with this conclusion, *tel1-400* was unable to improve the genotoxin resistance of *sae2*Δ cells when Rad9 SCD phosphorylation was compromised (Fig. 5B). Notably, *rad9-7xA* cells themselves displayed detectable sensitivity to CPT at higher drug concentrations (Fig. 5B), indicating that Rad9 phosphorylation-dependent oligomerization contributes to resistance to CPT-induced DNA damage also in *SAE2* cells, although its contribution is less pronounced than that of Sae2, which also promotes DSB processing and checkpoint downregulation. Although *rad9-7xA* slightly reduced the DNA damage sensitivity of *sae2*Δ cells, likely by attenuating checkpoint signaling, *tel1-400 rad9-7xA sae2*Δ cells displayed DNA damage sensitivity comparable to that of *rad9-7xA sae2*Δ cells and greater than that of *tel1-400 sae2*Δ cells (Fig. 5B). Because *tel1-400* retains the ability to suppress the DNA damage sensitivity of *sae2*Δ *rad53-K227A* cells (Fig. 3B), the checkpoint defect caused by *rad9-7xA* per se cannot account for the failure of *tel1-400* to suppress *rad9-7xA sae2*Δ cells. Thus, these findings indicate that Rad9 oligomerization driven by SCD phosphorylation is specifically required for *tel1-400*-dependent rescue.

To test more directly whether Rad9 self-association is required both for its end-tethering function and for *tel1-400*-dependent rescue, we exploited the *rad9-*Δ*BRCT::FKBP* allele, in which the C-terminal BRCT repeats are replaced by an FKBP tag. Because the BRCT repeats are required for Rad9 oligomerization [53], Rad9-ΔBRCT::FKBP is defective in self-association; however, its dimerization can be induced by the small molecule AP20187 [54]. This chemically induced dimerization promotes chromatin association and partially restores checkpoint signaling [29, 55].

Cultures were pre-incubated with AP20187 for 6 h. HO expression was then induced by galactose addition in cells arrested either in G1 with α-factor (Fig. 5C) or in G2 with nocodazole (Supplementary Fig. S8A), and cells were maintained under the same arrest conditions throughout the experiment. G1-arrested *rad9-*Δ*BRCT::FKBP* cells exhibited an increased fraction of cells with two LacI-GFP foci after HO induction, and this fraction decreased upon AP20187 treatment (Fig. 5C), indicating that chemically induced Rad9 dimerization improves DSB end-tethering. Similar results were obtained in G2-arrested cells (Supplementary Fig. S8A).

Because the MRX complex contributes to DSB end-tethering [52], we asked whether the AP20187-induced enhancement of end-tethering was mediated by increased MRX retention at DSB ends. To address this possibility, we monitored Rad50 association with the HO-induced DSB. AP20187 treatment did not increase Rad50 association with the break in *rad9-*Δ*BRCT::FKBP* cells (Supplementary Fig. S8B), suggesting that the improvement in DSB end-tethering does not result from increased MRX retention. This finding supports the idea that Rad9 self-association actively contributes to maintaining DSB-end proximity.

Notably, *tel1-400* suppressed the end-tethering defect of G1- and G2-arrested *rad9-*Δ*BRCT::FKBP sae2*Δ cells only upon AP20187 treatment (Fig. 5C and Supplementary Fig. S8A), supporting the requirement for Rad9 self-association in *tel1-400*-dependent rescue. By contrast, in *rad9-*Δ*BRCT::FKBP sae2*Δ cells carrying wild-type *TEL1*, AP20187 reduced end separation only to the level observed in *sae2*Δ cells (Fig. 5C and Supplementary Fig. S8A), indicating that enforced Rad9 dimerization compensates for loss of the Rad9 BRCT domains but, in the absence of *tel1-400*, does not suppress the end-tethering defect caused by *SAE2* deletion.

Consistent with these findings, *tel1-400* improved the DNA damage resistance of *rad9-*Δ*BRCT::FKBP sae2*Δ cells only upon AP20187 treatment (Fig. 5D). AP20187 also slightly reduced the DNA damage sensitivity of *rad9*-Δ*BRCT::FKBP* cells (Fig. 5D), further supporting a contribution of Rad9 self-association to resistance to DNA damage in otherwise *SAE2*-proficient cells. AP20187 also restored the resistance of *rad9*-Δ*BRCT::FKBP sae2*Δ cells to that of *sae2*Δ cells (Fig. 5D). Thus, enforced Rad9 dimerization suppresses the additional sensitivity caused by loss of the BRCT domains but cannot overcome the *sae2*Δ-associated sensitivity without *tel1-400*. Together, these results show that Rad9 self-association contributes to DSB end-tethering and is required for *tel1-400*-dependent restoration of both end-tethering and genotoxin resistance.

### The *tel1-400* allele promotes the accumulation of NCO and CO products while reducing SSA in *sae2*Δ cells

Maintenance of DSB ends in close proximity facilitates repair by pathways that require coordination between the two DNA ends, including NHEJ and HR. Because *sae2*Δ cells display elevated NHEJ, likely as a consequence of reduced resection [27], the increased DNA damage resistance of *tel1-400 sae2*Δ cells is unlikely to result from enhanced NHEJ. Consistently, *tel1-400* still suppressed the DNA damage sensitivity of *sae2*Δ *lig4*Δ and *sae2*Δ *ku70*Δ cells, which are defective in NHEJ due to loss of DNA ligase IV or the Ku70/Ku80 heterodimer, respectively (Supplementary Fig. S9A and B).

We therefore examined whether *tel1-400* affects DSB repair by HR. During canonical HR, a 3′ ssDNA tail invades a homologous duplex to form a D-loop, and subsequent processing of recombination intermediates can generate either noncrossover (NCO) or crossover (CO) products. Alternatively, in synthesis-dependent strand annealing (SDSA), the extended invading strand is displaced from the D-loop and anneals to complementary ssDNA generated at the second DSB end, producing exclusively NCOs (Supplementary Fig. S10A) [1].

**Figure 6.**
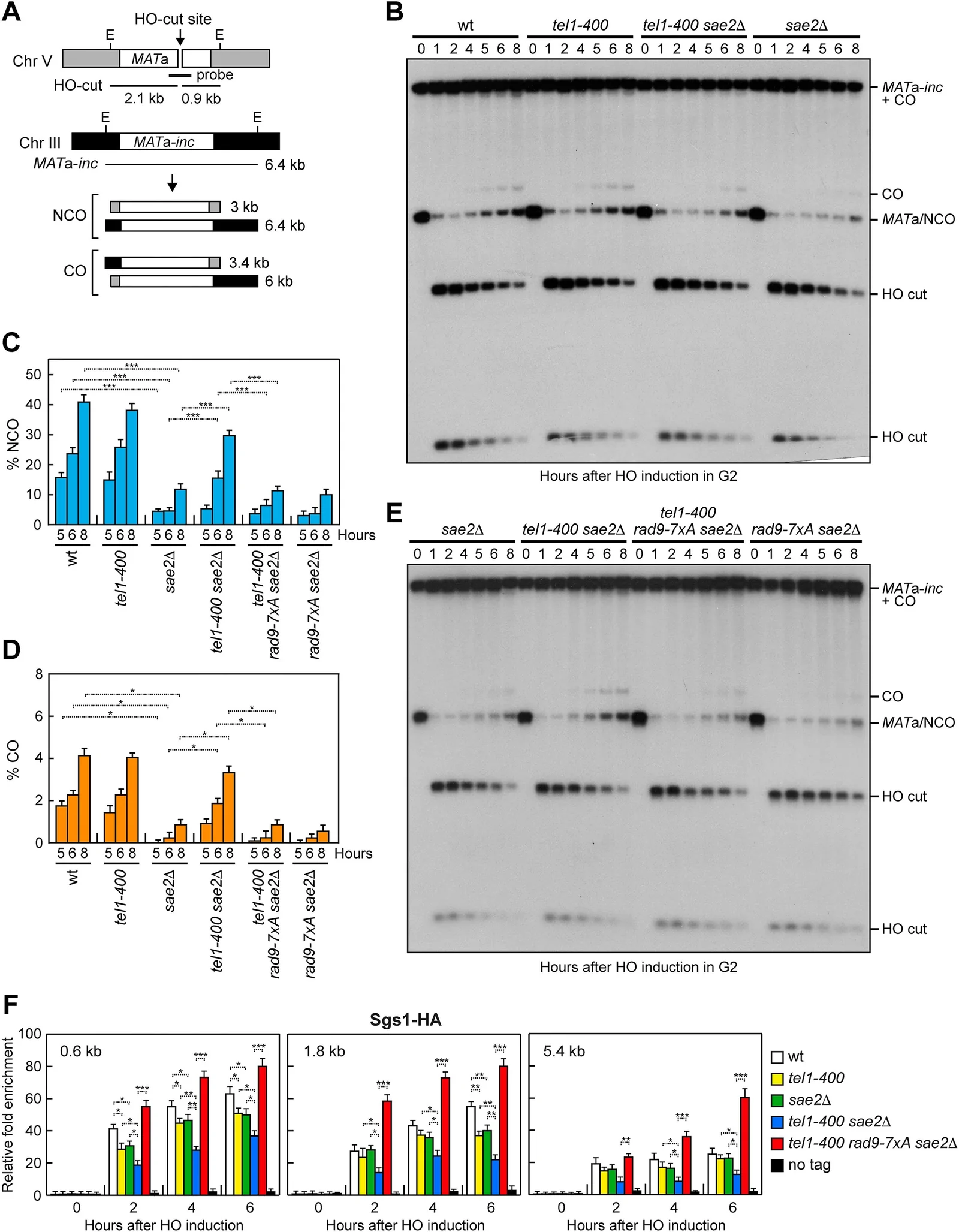
*tel1-400* increases NCO and CO product accumulation in *sae2*Δ cells. (**A**) Assay to monitor ectopic HR repair. HO induction generates a DSB at the *MAT*a sequence on chromosome V, whereas the homologous *MAT*a*-inc* region on chromosome III cannot be cut by HO and is used as a donor for HR-mediated repair, yielding NCO and CO products. E, EcoRI. (**B**) Southern blot analysis of HR products. YEPR cultures were arrested in G2 with nocodazole and shifted to YEPRG at time zero in the presence of nocodazole. EcoRI-digested genomic DNA was analyzed by Southern blot using a *MAT*a probe. (**C** and **D**) Densitometric analysis of NCO (C) and CO (D) band signals. Bars represent the mean ± s.d. from three independent experiments; ***P < 0.005; *P < 0.05 (unpaired two-tailed Student’s *t*-test). (**E**) Southern blot analysis of HR products performed as in panel (B). (**F**) ChIP-qPCR analysis of Sgs1 occupancy at an HO-induced DSB. YEPR cultures of JKM139-derived strains were shifted to YEPRG to induce HO expression. Relative Sgs1–HA enrichment at the indicated distance from the HO cut site was measured after anti-HA ChIP followed by qPCR. Bars represent the mean ± s.d. from three independent experiments; ***P < 0.005, **P < 0.01, *P < 0.05 (unpaired two-tailed Student’s *t*-test).

To monitor HR outcomes, we used a DSB-induced ectopic gene conversion assay in the tGI354 background, in which galactose-induced HO endonuclease generates a DSB at *MAT*a on chromosome V that is repaired using an uncleavable *MAT*a*-inc* donor on chromosome III (Fig. 6A) [56, 57]. This assay yields both NCO and CO products, with COs representing approximately 5% of total repair events. Galactose was maintained throughout the experiment to ensure continuous cleavage of any HO sites reconstituted by end joining. Under these conditions, *tel1-400 sae2*Δ cells accumulated both the 3.0 kb NCO and the 3.4 kb CO products to higher levels than *sae2*Δ cells (Fig. 6B–D), indicating that *tel1-400* increases both NCO and CO formation in the absence of Sae2. Although NCO and CO products accumulated to higher levels in *tel1-400 sae2*Δ cells, their formation kinetics were similar to those observed in *sae2*Δ cells, consistent with the finding that *tel1-400* does not suppress the DSB resection defect of *sae2*Δ cells.

Introduction of the *rad9-7xA* allele, which impairs Rad9 oligomerization and DSB end-tethering, reduced both NCO and CO product formation in *tel1-400 sae2*Δ cells (Fig. 6C–E), indicating that *tel1-400* requires Rad9 phosphorylation-dependent oligomerization to promote the accumulation of strand-invasion-dependent HR products in *sae2*Δ cells. Because formation of both NCO and CO products requires productive coordination between the two DSB ends, including capture or engagement of the second end after donor invasion [58], improved Rad9-dependent end-tethering may provide an architectural framework that favors completion of both repair outcomes.

Rad9 may also influence crossover-associated repair by limiting the recruitment of the anti-crossover helicases Sgs1 and Mph1 at DSBs [59]. Sgs1 and Mph1 promote strand rejection and restrict productive D-loop extension [56, 59, 60], and loss of *SGS1* and/or *MPH1* increases CO formation and partially restores CO accumulation in *rad9*Δ cells [59]. Because the *sgs1*Δ *sae2*Δ combination was synthetically lethal [61] and this lethality was not rescued by *tel1-400* (Supplementary Fig. S11), we could not directly test the effect of *SGS1* deletion on CO formation in *tel1-400 sae2*Δ cells. We therefore examined Sgs1 association with DSBs. Sgs1 enrichment at the break was slightly reduced in *tel1-400* and *sae2*Δ single mutants but markedly decreased in the *tel1-400 sae2*Δ double mutant (Fig. 6F). Importantly, *rad9-7xA* increased Sgs1 association in *tel1-400 sae2*Δ cells to levels higher than those observed in wild-type cells (Fig. 6F), indicating that Rad9 phosphorylation-dependent oligomerization limits Sgs1 association with DSBs. Together, these results indicate that, in *sae2*Δ cells, *tel1-400* promotes NCO and CO accumulation and limits Sgs1 association with DSBs through Rad9 phosphorylation-dependent oligomerization.

**Figure 7.**
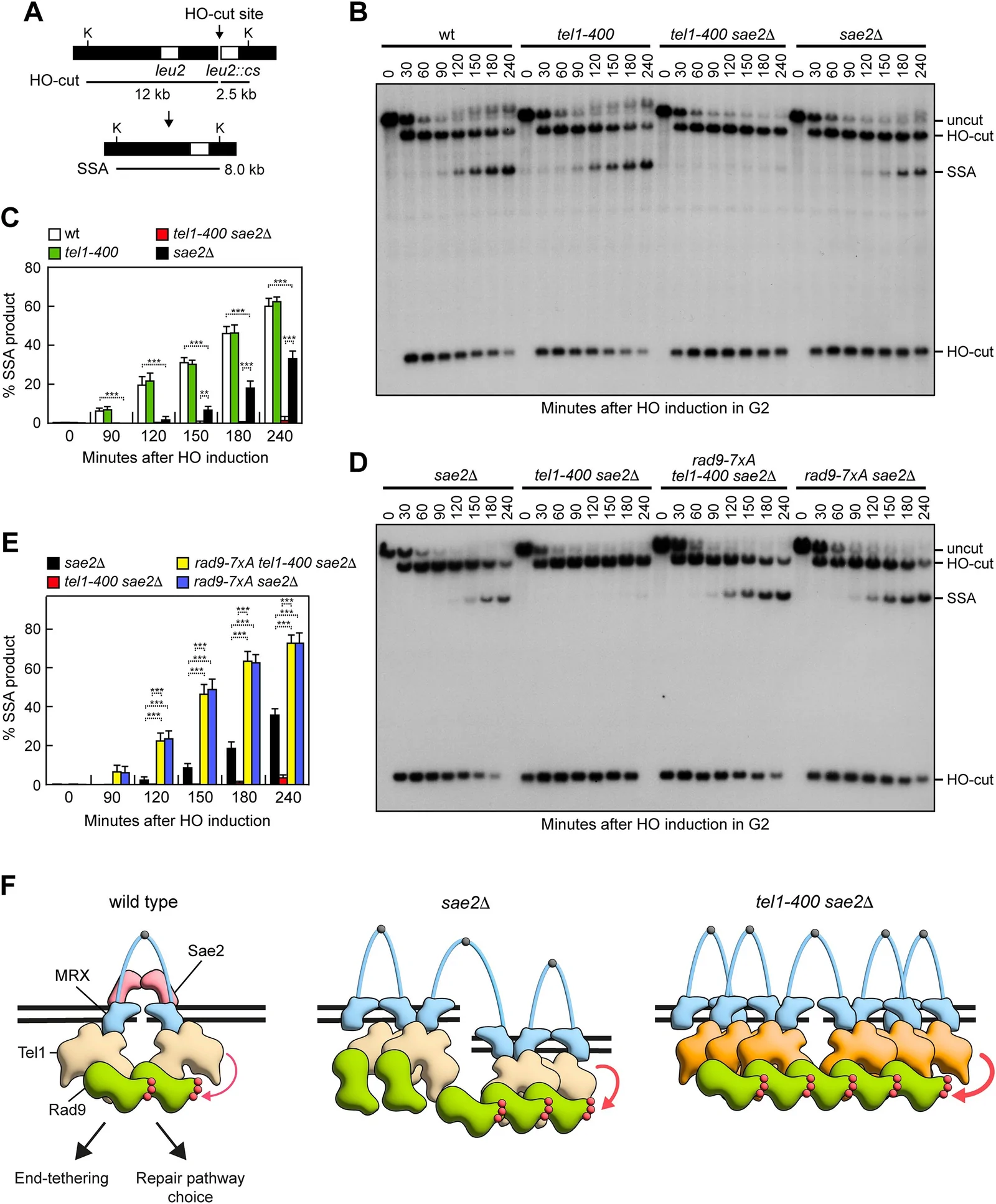
*tel1-400* decreases SSA in *sae2*Δ cells in a Rad9 oligomerization-dependent manner. (**A**) Assay to monitor SSA repair. HO induction generates a DSB between two homologous *leu2* repeats separated by 4.6 kb; K, KpnI. (**B**) Southern blot analysis of SSA products. YEPR cultures were arrested in G2 with nocodazole and transferred to YEPRG at time zero in the presence of nocodazole. KpnI-digested genomic DNA was analyzed by Southern blot using a *LEU2* probe. HO cutting yields 2.5 and 12 kb fragments (HO-cut), whereas SSA repair generates an 8 kb fragment (SSA). (**C**) Densitometric analysis of the SSA band signals. Bars represent the mean ± s.d. from three independent experiments; ***P < 0.005, **P < 0.01 (unpaired two-tailed Student’s *t*-test). (**D**) Southern blot analysis of SSA products performed as in panel (B). (**E**) Densitometric analysis of the SSA band signals. Bars represent the mean ± s.d. from three independent experiments; ***P < 0.005 (unpaired two-tailed Student’s *t*-test). (**F**) Model for Tel1–Rad9 control of DSB-end architecture and repair pathway choice. In wild-type cells, MRX recruits and activates Tel1 at DNA ends, promoting Rad9 phosphorylation (red dots) and Rad9 self-association into higher-order assemblies that help maintain DSB-end proximity and influence repair pathway choice. In *sae2*Δ cells, DSB end-tethering is partially impaired, while MRX–Tel1 and Rad9 persist at the break and Rad9 phosphorylation is modestly increased. Under these conditions, Rad9 oligomerization still contributes to residual DSB-end proximity. In *tel1-400 sae2*Δ cells, increased Tel1 retention at DNA ends and enhanced Rad9 phosphorylation reinforce Rad9 oligomerization at DSBs, thereby improving DSB-end proximity, promoting strand-invasion-dependent HR, and further inhibiting SSA. Gray dots indicate Zn^2+^ ions.

We next asked whether *tel1-400* also affects SSA, a strand-invasion-independent HR subpathway that repairs DSBs between direct repeats by annealing complementary ssDNA exposed by resection (Supplementary Fig. S10B) [62]. This question was prompted by our finding that *tel1-400* reduces Sgs1 association with DSBs in a Rad9 oligomerization-dependent manner and by previous evidence that loss of Sgs1 severely impairs SSA in *sae2*Δ *rad9*Δ cells without further affecting bulk resection [29]. We therefore monitored SSA product formation in a strain carrying tandem *LEU2* repeats with an HO recognition site adjacent to one repeat (Fig. 7A) [63]. As expected, *sae2*Δ cells accumulated fewer SSA products than wild-type cells (Fig. 7B and C), likely because of combined defects in resection and end-tethering [31]. The presence of *tel1-400* further reduced SSA product accumulation in *sae2*Δ cells (Fig. 7B and C), indicating that *tel1-400* inhibits SSA in the absence of Sae2. This reduction cannot be attributed to impaired end resection, because ssDNA intermediates accumulated with similar kinetics in *sae2*Δ and *tel1-400 sae2*Δ cells (Fig. 3C and D). Notably, *rad9-7xA* increased SSA product formation in *sae2*Δ cells and restored SSA products in *tel1-400 sae2*Δ cells to levels comparable to those observed in *rad9-7xA sae2*Δ cells (Fig. 7D and E). These findings indicate that Rad9 phosphorylation-dependent oligomerization restrains SSA in the absence of Sae2 and that *tel1-400* enhances this Rad9-dependent inhibitory effect.

## Discussion

Loss of Sae2 creates a multifaceted defect in the DSB response, in which impaired DNA-end processing is coupled to sustained checkpoint signaling. Accordingly, improved DNA damage resistance in *sae2*Δ cells has generally been associated either with attenuation of checkpoint activation through reduced Tel1/Rad53 signaling or with restoration of resection through relief of Rad9-mediated inhibition of Exo1 and Sgs1–Dna2 activities [20, 21, 25, 26, 28].

Here, we identify a Tel1 FAT-domain variant, Tel1-400, that suppresses the DNA damage sensitivity of *sae2*Δ cells without attenuating checkpoint activation or restoring either resection or Mre11 nuclease-dependent DNA-end processing. Instead, *tel1-400* alleviates the defect of *sae2*Δ cells in maintaining DSB ends in close proximity, indicating that Tel1 can promote DNA damage resistance through a mechanism linked to DSB-end architecture. The major phenotypes associated with the strains analyzed in this study are summarized in Supplementary Table S4.

Tel1-400-mediated rescue depends on Rad9 oligomerization. Tel1-400 neither reduces end separation nor improves genotoxin resistance in *rad9*Δ *sae2*Δ cells. Moreover, the oligomerization-defective *rad9-7xA* mutant abolishes *tel1-400*-dependent rescue, whereas chemically induced Rad9 dimerization restores DSB end-tethering and enables *tel1-400* to suppress both end separation and genotoxin sensitivity in *sae2*Δ cells.

Rad9 also contributes to DSB-end organization independently of *tel1-400*, as *RAD9* deletion or impaired Rad9 oligomerization increases end separation in wild-type cells and further exacerbates the tethering defect of *sae2*Δ cells. This suggests that Rad9 accumulation in *sae2*Δ cells helps maintain residual DSB-end proximity. Tel1-400 may suppress this defect by favoring oligomerization-dependent Rad9 assemblies at DSBs.

Because Tel1-400 association with DSBs depends on MRX, which persists at DNA ends in *sae2*Δ cells, our data do not establish whether the Tel1–Rad9 axis functions as an independent tethering module or instead reinforces MRX-dependent tethering under physiological conditions. However, chemically induced Rad9 dimerization promotes DSB end-tethering without increasing MRX retention at DSBs, supporting an architectural function for Rad9 at DSBs that is potentiated by Tel1-400 in Sae2-defective cells.

How does Tel1-400 enhance Rad9-dependent DSB end-tethering? Although Tel1-400 does not display increased intrinsic kinase activity *in vitro*, suppression of *sae2*Δ requires its catalytic activity. Notably, *tel1-kd* cells do not show a detectable end-tethering defect [33, 64]. However, because Rad9 can be phosphorylated by both Tel1 and Mec1 [14], Mec1-dependent phosphorylation may compensate for the loss of Tel1 catalytic activity in *tel1-kd* cells, although this possibility cannot be directly tested because cells simultaneously defective for Mec1 and Tel1 undergo senescence [65]. *In vivo*, Tel1-400 accumulates more strongly at DSBs than wild-type Tel1 and promotes increased DNA-damage-induced Rad9 phosphorylation. Thus, rather than increasing the intrinsic catalytic activity of Tel1, the *tel1-400* mutation may enhance local Tel1 signaling at DSBs, thereby favoring BRCT–SCD-dependent Rad9 oligomerization and DSB end-tethering.

The increased accumulation of Tel1-400 at DSBs is unlikely to reflect a simple gain in intrinsic DNA-binding activity, because the *tel1-400* substitutions do not map to known DNA-contacting surfaces of Tel1 or other PIKK-family members. Instead, their distribution across N-FAT and M-FAT points to altered FAT-dependent allosteric regulation. In PIKK-family kinases, the FAT domain forms an extended helical scaffold around the kinase domain and contributes to conformational changes that couple DNA-end engagement to kinase-domain positioning and substrate access [47, 48]. The positions of the *tel1-400* substitutions are consistent with altered FAT-dependent coupling: I1765 lies within N-FAT, close to an interface with the kinase domain that is remodeled in more open, active-like Tel1 conformations, whereas K1879 and N2004 map within M-FAT, where our CG-MD simulations revealed altered dynamic coordination with N-FAT relative to wild-type Tel1. Thus, rather than disrupting a single local contact, the *tel1-400* substitutions appear to alter coordinated FAT-domain motions and their coupling to the kinase domain.

This interpretation is consistent with structural studies of other PIKK-family kinases, in which FAT-domain rearrangements accompany transitions between inactive and active states. In human mTORC1, activation involves coordinated motions within the FAT domain that propagate toward the C-FAT region, including the LID and HEAT-repeat domain (HRD) elements, and toward the kinase N-lobe [66]. Although equivalent conformational transitions have not been fully defined for Tel1, comparisons among partially resolved Tel1 conformations indicate that more open kinase configurations are accompanied by movements within M-FAT and repositioning of the LID and HRD regions and the kinase N-lobe [37, 48]. The altered dynamic coordination observed in the Tel1-400 model is consistent with a conformational ensemble that could stabilize Tel1-400 engagement at MRX-bound DNA ends. This could enhance phosphorylation of nearby chromatin-associated substrates, including Rad9, and promote higher-order Rad9 assemblies through BRCT–SCD interactions, thereby contributing to DSB-end architecture. In otherwise wild-type cells, the more limited association of Rad9 with DSBs may constrain the consequences of this altered Tel1 behavior. By contrast, in *sae2*Δ cells, where Rad9 association with DSBs is already increased, prolonged Tel1-400 engagement at DNA ends may favor higher-order Rad9 assembly that compensates for defective DSB end-tethering (Fig. 7F).

Notably, *tel1-400* does not increase bulk Rad9 recruitment at DSBs in either wild-type or *sae2*Δ cells, indicating that it alters the functional state of Rad9 rather than its overall recruitment. A similar uncoupling between recruitment and higher-order function has been reported for 53BP1, the mammalian ortholog of Rad9, as an oligomerization-defective variant impairs NHEJ-mediated fusion of dysfunctional telomeres without preventing 53BP1 accumulation at these sites [67].

By maintaining DSB-end proximity, the Tel1–Rad9 axis may limit inappropriate end capture and facilitate proper repair synapsis. In *sae2*Δ cells, *tel1-400* increases both NCO and CO recombination products while strongly reducing SSA products, despite resection kinetics similar to those observed in *sae2*Δ cells. These opposite effects of Tel1-400 on strand-invasion-dependent HR and SSA are abolished by the *rad9-7xA* allele, indicating that Rad9 phosphorylation-dependent oligomerization differentially influences the use of resected substrates by distinct HR pathways.

The increased accumulation of NCO and CO products is consistent with improved DSB end-tethering. Strand-invasion-dependent HR requires coordination between the two DSB ends, including capture or engagement of the second end after donor invasion. Enhanced Rad9-dependent end-tethering in *tel1-400 sae2*Δ cells may therefore help maintain coordination between the donor-invasion intermediate and the second DSB end, facilitating completion of the HR reaction.

Elevated CO formation may also reflect reduced dismantling of D-loops. The Sgs1 and Mph1 helicases counteract CO formation by promoting strand rejection and limiting D-loop extension, thereby decreasing the stability of strand-invasion intermediates [56, 68–70]. Rad9 has been proposed to favor CO-prone repair by restricting Sgs1 and Mph1 recruitment and/or activity at DSBs, allowing recombination intermediates to persist and mature [59]. Consistent with this scenario, the combination of *tel1-400* and *SAE2* loss markedly reduces Sgs1 association with DSBs through a mechanism requiring Rad9 phosphorylation-dependent oligomerization. Together, these findings suggest that Tel1-400 promotes a Rad9 oligomerization-dependent repair architecture that supports coordination between the donor-invasion intermediate and the second DNA end, thereby favoring both NCO and CO formation. Reduced Sgs1 recruitment may further contribute to elevated CO formation in *sae2*Δ cells.

The Rad9 oligomerization-dependent architecture promoted by *tel1-400* may also contribute to SSA inhibition in *sae2*Δ cells. Indeed, *rad9-7xA* restores SSA in *tel1-400 sae2*Δ cells, indicating that SSA inhibition requires Rad9 phosphorylation-dependent oligomerization. The marked reduction in SSA is unlikely to reflect defective DSB resection, because resection is not detectably reduced compared with *sae2*Δ cells. Although diversion of repair intermediates toward strand-invasion-dependent HR may contribute to the SSA defect, it is unlikely to provide the sole explanation. Tel1-400 may therefore also limit SSA through an additional Rad9 oligomerization-dependent effect on the use of resected DNA ends.

Previous work showed that loss of *SGS1* strongly decreases SSA in *sae2*Δ *rad9*Δ cells without impairing DSB resection [29], indicating that Sgs1 promotes efficient SSA downstream of resection, at least in this repair context. The reduced Sgs1 association observed in *tel1-400 sae2*Δ cells may therefore have two consequences: it may favor the persistence of strand-invasion intermediates, thereby contributing to CO formation, while simultaneously limiting Sgs1-dependent remodeling events that facilitate SSA. One possibility is that Sgs1 increases the accessibility of complementary ssDNA regions to Rad52-mediated annealing by removing secondary structures, protein-bound obstacles, or competing recombination intermediates that would otherwise hinder productive annealing between direct repeats.

Although Rad9 and metazoan 53BP1 share limited primary sequence conservation, the requirement for higher-order assembly appears to be functionally conserved. 53BP1 oligomerization is required for synapsis and end joining in several contexts, including immunoglobulin class-switch recombination, long-range end joining, and telomere fusions [67, 71–75]. These findings suggest that 53BP1 self-assembly may facilitate the clustering and/or synapsis of distal DNA ends. In addition to its roles in end joining, 53BP1 can influence homology-directed repair outcomes, as depletion of 53BP1 or saturation of its chromatin-binding capacity shifts repair from RAD51-dependent gene conversion toward RAD52-dependent SSA [76]. Together, these observations support the idea that higher-order 53BP1 assemblies contribute to DSB-end organization and repair-pathway choice, paralleling the requirement for Rad9 self-association identified here.

Overall, our findings identify a Tel1–Rad9 axis in which Tel1-dependent Rad9 phosphorylation and self-association contribute to DSB-end organization and repair-pathway choice. The increased persistence of Tel1-400 at DSBs potentiates this activity in *sae2*Δ cells, favoring strand-invasion-dependent HR while limiting SSA, with potential parallels to oligomerization-dependent 53BP1 functions in mammals.

## Supplementary Material

gkag919_Supplemental_Files

## Data Availability

The data underlying this article are available in the article and in its online supplementary material.
