# Supplementary material for: A Tel1–Rad9 axis links DNA double-strand break end-tethering to repair pathway choice
Source: Nucleic Acids Res. 2026 Sep 18;54(17):gkag919. doi: 10.1093/nar/gkag919 (PMC13586538; doi:10.1093/nar/gkag919)
Supplement: Supplementary file 1 [file gkag919_supplemental_files.zip › Supplementary Data.pdf]

Supplementary Table S1. ***Saccharomyces cerevisiae*** **strains used in this study.**

Strain	Relevant genotype	Source
W303	*MAT*a/α *ade2-1 can1-100 his3-11,15 leu2-3,112 trp1-1 ura3-1 rad5-535*	
YLL1069.3	W303 *MAT*a *sae2Δ::KANMX*	1
DMP6268.4A	W303 *MAT*a *tel1-hy394::LEU2 sae2Δ::KANMX*	This study
YLL4385.6	W303 *MAT*a *tel1-400::LEU2 sae2Δ::HIS3*	This study
YLL4994.3	W303 *MAT*a *tel1-I1765V::LEU2 sae2Δ::KANMX*	This study
YLL4995.4	W303 *MAT*a *tel1-K1879N::LEU2 sae2Δ::KANMX*	This study
YLL4996.2	W303 *MAT*a *tel1-N2004S::LEU2 sae2Δ::KANMX*	This study
YLL4997.2	W303 *MAT*a *tel1-I1765V,K1879N::LEU2 sae2Δ::KANMX*	This study
YLL4998.14	W303 *MAT*a *tel1-I1765V,N2004S::LEU2 sae2Δ::KANMX*	This study
YLL4999.9	W303 *MAT*a *tel1-K1879N,N2004S::LEU2 sae2Δ::KANMX*	This study
DMP8262.4	W303 *MAT*a/α *TEL1/TEL1 SAE2/SAE2*	This study
DMP8263.2	W303 *MAT*a/α *tel1-400::HPHMX/tel1-400::HPHMX sae2Δ::KANMX/ sae2Δ::KANMX*	This study
DMP8264.1	W303 *MAT*a/α *TEL1/tel1-400::HPHMX sae2Δ::KANMX/ sae2Δ::KANMX*	This study
DMP8265.1	W303 *MAT*a/α *TEL1/TEL1 sae2Δ::KANMX/ sae2Δ::KANMX*	This study
YLL490.4	W303 *MAT*a *mec1Δ::HIS3 sml1Δ::KANMX*	2
DMP4690.9A	W303 *MAT*a *TEL1-3HA mec1Δ::HIS3 sml1Δ::KANMX*	2
YLL1975.1	W303 *MAT*a *tel1-hy394-3HA::LEU2 mec1Δ::HIS3 sml1Δ::KANMX*	2
DMP8266.9A	W303 *MAT*a *tel1-400-3HA::LEU2 mec1Δ::HIS3 sml1Δ::KANMX*	This study
DMP8281.6B	W303 *MAT*a *tel1-hy394::LEU2*	This study
YLL5000.5	W303 *MAT*a *tel1-400::HPHMX*	This study
DMP8267.2B	W303 *MAT*α *mec1Δ::HIS3 sml1Δ::KANMX*	This study
YLL5025.1	W303 *MAT*a *tel1-hy394::LEU2 mec1Δ::HIS3 sml1Δ::KANMX*	2
DMP8267.3B	W303 *MAT*a *tel1-400::LEU2 mec1Δ::HIS3 sml1Δ::KANMX*	This study
DMP8268.10B	W303 *MAT*α *tel1-400-kd::LEU2 sae2Δ::KANMX*	This study
DMP7632.4B	W303 *MAT*α *tel1-kd::LEU2 sae2Δ::KANMX*	This study
YLL5001.5	W303 *MAT*a *tel1-400-kd::LEU2*	This study
YLL4580.4	W303 *MAT*a *tel1-kd::LEU2*	This study
DMP8269.10B	W303 *MAT*a *tel1-400::LEU2 sae2Δ::HIS3 rad53-K227A::KANMX*	This study
DMP8269.4A	W303 *MAT*a *sae2Δ::HIS3 rad53-K227A::KANMX*	This study
DMP8269.11B	W303 *MAT*α *tel1-400::LEU2 rad53-K227A::KANMX*	This study

DMP8269.5C	W303 *MAT*a *rad53-K227A::KANMX*	This study
DMP8270.9A	W303 *MAT*a *tel1-400::LEU2 sae2Δ::HIS3 mre11-H125N::HPHMX*	This study
DMP8270.4B	W303 *MAT*α *mre11-H125N::HPHMX*	This study
DMP8270.10B	W303 *MAT*a *sae2Δ::HIS3 mre11-H125N::HPHMX*	This study
DMP8270.2A	W303 *MAT*a *tel1-400::LEU2 mre11-H125N::HPHMX*	This study
DMP8271.9A	W303 *MAT*a *tel1-400::LEU2 sae2Δ::KANMX rad9Δ::URA3*	This study
DMP8271.13A	W303 *MAT*a *sae2Δ::KANMX rad9Δ::URA3*	This study
DMP8271.12D	W303 *MAT*α *tel1-400::LEU2 rad9Δ::URA3*	This study
DMP8271.11D	W303 *MAT*α *rad9Δ::URA3*	This study
DMP8272.6A	W303 *MAT*a *tel1-400::LEU2 sae2Δ::KANMX rad9-7xA::HPHMX*	This study
DMP8272.7A	W303 *MAT*a *sae2Δ::KANMX rad9-7xA::HPHMX*	This study
DMP8272.12C	W303 *MAT*a *rad9-7xA::HPHMX*	This study
YLL5002.13	W303 *MAT*a *rad9-ΔBRCT::FKBP::KANMX*	This study
DMP8273.4A	W303 *MAT*α *tel1-400::LEU2 rad9-ΔBRCT::FKBP::KANMX sae2Δ::HIS3*	This study
DMP8273.11D	W303 *MAT*α *rad9-ΔBRCT::FKBP::KANMX sae2Δ::HIS3*	This study
DMP8274.3C	W303 *MAT*α *tel1-400::LEU2 sae2Δ::HIS3 lig4Δ::KANMX*	This study
DMP8274.3A	W303 *MAT*a *sae2Δ::HIS3 lig4Δ::KANMX*	This study
DMP8274.1A	W303 *MAT*a *tel1-400::LEU2 lig4Δ::KANMX*	This study
DMP8274.11B	W303 *MAT*a *lig4Δ::KANMX*	This study
DMP8275.12A	W303 *MAT*a *tel1-400::LEU2 sae2Δ::KANMX ku70Δ::HIS3*	This study
DMP8275.1C	W303 *MAT*a *sae2Δ::KANMX ku70Δ::HIS3*	This study
DMP8276.22B	W303 *MAT*a *tel1-400::HPHMX ku70Δ::HIS3*	This study
DMP8276.22A	W303 *MAT*a *ku70Δ::HIS3*	This study
JKM139	*MAT*a *hmlΔ::ADE1 hmrΔ::ADE1 ade1-100 lys5 leu2-3, 112 trp::hisG ura3-52 ho ade3::GAL-HO site*	3
YLL1523.3	JKM139 *MAT*a *sae2Δ::KANMX*	1
YLL5002.9	JKM139 *MAT*a *tel1-400::LEU2*	This study
DMP8277.5C	JKM139 *MAT*a *tel1-400::LEU2 sae2Δ::KANMX*	This study
YLL5070.1	JKM139 *MAT*a *rad9-7xA::HPHMX*	This study
YLL3222.6	JKM139 *MAT*a *TEL1-3HA::NAT*	4
YLL5003.19	JKM139 *MAT*a *tel1-400-3HA::LEU2::NATMX*	This study
DMP8278.11B	JKM139 *MAT*a *TEL1-3HA::NAT sae2Δ::KANMX*	This study
DMP8279.4A	JKM139 *MAT*a *tel1-400-3HA::LEU2::NATMX sae2Δ::KANMX*	This study
YLL5004.1	JKM139 *MAT*a *tel1-400-3HA::LEU2::NATMX mre11Δ::URA3*	This study

YLL5005.1	JKM139 *MAT*a *TEL1-3HA::NAT mre11Δ::URA3*	This study
DMP8279.3A	JKM139 *MAT*a *RAD9-3HA::TRP1*	This study
DMP8279.9D	JKM139 *MAT*a *RAD9-3HA::TRP1 tel1-400::LEU2*	This study
DMP8279.5D	JKM139 *MAT*a *RAD9-3HA::TRP1 sae2Δ::KANMX*	This study
DMP8279.8B	JKM139 *MAT*a *RAD9-3HA::TRP1 tel1-400::LEU2 sae2Δ::KANMX*	This study
DMP8280.3B	JKM139 *MAT*a *SGS1-3HA::URA3*	This study
DMP8280.8C	JKM139 *MAT*a *SGS1-3HA::URA3 tel1-400::LEU2*	This study
DMP8280.5A	JKM139 *MAT*a *SGS1-3HA::URA3 sae2Δ::KANMX*	This study
DMP8280.5C	JKM139 *MAT*a *SGS1-3HA::URA3 tel1-400::LEU2 sae2Δ::KANMX*	This study
DMP8330.6D	JKM139 *MATα SGS1-3HA::URA3 tel1-400::LEU2 sae2Δ::KANMX rad9-7xA::HPHMX*	This study
YLL5071.2	JKM139 *MAT*a *rad9-7xA-3HA::HPHMX::TRP1*	This study
YLL3501.1	JKM139 *MAT*a *RAD50-3HA::URA3*	5
YLL5072.1	JKM139 *MAT*a *RAD50-3HA::URA3 rad9-ΔBRCT::FKBP::HPHMX*	This study
YJK40.6	*MATΔ hmlΔ hmrΔ can1 lys5 ade2 leu2 trp1 ura3 his3 ade3:: GAL-HO VII::TRP1-HO LacI-GFP::URA3 LacO::LYS5 LacO::KanR*	6
YLL1709.11	YJK40.6 *sae2Δ::NATMX*	7
YLL5006.4	YJK40.6 *tel1-400::LEU2*	This study
YLL5007.22	YJK40.6 *tel1-400::LEU2 sae2Δ::NATMX*	This study
YLL5008.1	YJK40.6 *tel1-400::LEU2 rad9Δ::HPHMX sae2Δ::NATMX*	This study
YLL5009.1	YJK40.6 *rad9Δ::HPHMX sae2Δ::NATMX*	This study
YLL5010.3	YJK40.6 *tel1-400::LEU2 rad9Δ::HPHMX*	This study
YLL5011.1	YJK40.6 *rad9Δ::HPHMX*	This study
YLL5012.1	YJK40.6 *tel1-400::LEU2 rad9-7xA::HPHMX sae2Δ::NATMX*	This study
YLL5013.3	YJK40.6 *rad9-7xA::HPHMX sae2Δ::NATMX*	This study
YLL5014.1	YJK40.6 *tel1-400::LEU2 rad9-7xA::HPHMX*	This study
YLL5015.1	YJK40.6 *rad9-7xA::HPHMX*	This study
YLL5016.2	YJK40.6 *rad9-ΔBRCT::FKBP::HPHMX*	This study
YLL5017.4	YJK40.6 *tel1-400::LEU2 rad9-ΔBRCT::FKBP::HPHMX sae2Δ::NATMX*	This study
YLL5018.2	YJK40.6 *rad9-ΔBRCT::FKBP::HPHMX sae2Δ::NATMX*	This study
tGI354	*ho hmlΔ::ADE1 MATa-inc hmrΔ::ADE1 ade1 leu2-3;112 lys5 trp1::hisG ura3-52 ade3::GAL::HO arg5,6::MATa::HPHMX*	8
YLL3914.2	tGI354 *sae2Δ::NATMX*	4
YLL5019.3	tGI354 *tel1-400::LEU2*	This study
YLL5020.4	tGI354 *tel1-400::LEU2 sae2Δ::NATMX*	This study
YLL5073.12	tGI354 *tel1-400::LEU2 sae2Δ::NATMX rad9-7xA::KANMX*	This study

YLL5074.4	tGI354 *sae2Δ::NATMX rad9-7xA::KANMX*	This study
YMV45	*ho hml::ADE1 mata::hisG hmr::ADE1 leu2::leu2(Asp718-SalI)-URA3*-pBR332-*MATa ade3::GAL::HO ade1 lys5 ura3-52 trp1::hisG*	9
YLL1621.9	YMV45 *sae2Δ::KANMX*	1
YLL5021.1	YMV45 *tel1-400::HPHMX*	This study
YLL5022.4	YMV45 *tel1-400::HPHMX sae2Δ::KANMX*	This study
YLL5075.5	YMV45 *tel1-400::NATMX sae2Δ::KANMX rad9-7xA::HPHMX*	This study
YLL5076.2	YMV45 *sae2Δ::KANMX rad9-7xA::HPHMX*	This study

References

1 Gobbini E, Villa M, Gnugnoli M, Menin L, Clerici M and Longhese MP. Sae2 Function at DNA double-strand breaks is bypassed by dampening Tel1 or Rad53 activity. PLoS Genet. 2015; 11: e1005685. doi: 10.1371/journal.pgen.1005685.

2 Baldo V, Testoni V, Lucchini G and Longhese MP. Dominant *TEL1-hy* mutations compensate for Mec1 lack of functions in the DNA damage response. Mol. Cell. Biol. 2008; 28: 358-375. doi: 10.1128/MCB.01214-07.

3 Lee SE, Moore JK, Holmes A, Umezu K, Kolodner RD and Haber JE. *Saccharomyces* Ku70, mre11/rad50 and RPA proteins regulate adaptation to G2/M arrest after DNA damage. Cell 1998; 94: 399-409. doi: 10.1016/s0092-8674(00)81482-8.

4 Cassani C, Gobbini E, Vertemara J, Wang W, Marsella A, Sung P, Tisi R, Zampella G and Longhese MP. Structurally distinct Mre11 domains mediate MRX functions in resection, end-tethering and DNA damage resistance. Nucleic Acids Res. 2018; 46: 2990-3008. doi: 10.1093/nar/gky086.

5 Cassani C, Gobbini E, Wang W, Niu H, Clerici M, Sung P and Longhese MP. Tel1 and Rif2 regulate MRX functions in end-tethering and repair of DNA double-strand breaks. PLoS Biol. 2016; 14: e1002387. doi: 10.1371/journal.pbio.1002387.

6 Kaye JA, Melo JA, Cheung SK, Vaze MB, Haber JE and Toczyski DP. DNA breaks promote genomic instability by impeding proper chromosome segregation. Curr. Biol. 2004; 14: 2096-2106. doi: 10.1016/j.cub.2004.10.051.

7 Clerici M, Mantiero D, Lucchini G and Longhese MP. The *Saccharomyces cerevisiae* Sae2 protein promotes resection and bridging of double strand break ends. J. Biol. Chem. 2005; 280: 38631-38638. doi: 10.1074/jbc.M508339200.

8 Saponaro M, Callahan D, Zheng X, Krejci L, Haber JE, Klein HL and Liberi G. Cdk1 targets Srs2 to complete synthesis-dependent strand annealing and to promote recombinational repair. PLoS Genet. 2010; 6: e1000858. doi: 10.1371/journal.pgen.1000858.

9 Vaze MB, Pellicioli A, Lee SE, Ira G, Liberi G, Arbel-Eden A, Foiani M and Haber JE. Recovery from checkpoint-mediated arrest after repair of a double-strand break requires Srs2 helicase. Mol. Cell 2002; 10: 373-385. doi: 10.1016/s1097-2765(02)00593-2.

Supplementary Table S2. Oligonucleotides used for gene tagging and disruptions.

Name	Sequence (5'-3')
PRP330	TGAATAAAGAATGATGATCGCTGGCGTTTAACATCTAGCATATATCTG CAATAATTTATCACTCATCGATGAATTCGAGCTCG
PRP331	TGTAAGCCATTAGGTGTTTGTATGTGAGATGGTGACTGGTGAAGAAAA TGTGTATCTAAAGCGTACGCTGCAGGTCGAC
PRP2193	ATTAAGAGAATGCAGACAATTGACGCAAGTTGTACCTGCTCAGATCCG ATAAAACTCGACTTGAAAGAGAGAGAGAGAAGC
PRP2194	CGCGAAGGCAAGCCCTTGGTTATAAATAGGATATAATATAATATAGGG ATCAAGTACAAGTCGTTCATGGTGACACTTT
PRP171	CAATAGTGGAGAACTTAACGATCAAGTGGATGAAACAGGTTATACGT ACGCTGCAGGTCGAC
PRP172	ATTTAGCCTTTGGATGATTGGATCTTCTGACTTCTCAGATTCTAATCGA TGAATTCGAGCTCG
PRP730	ATGATATTACGGACAATGATATATACAACACTATTTCTGAGGTTAGAT CCGGTTCTGCTGCTAG
PRP731	TTAATCGTCCCTTTCTATCAATTATGAGTTTATATATTTTTATAATTCCT CGAGGCCAGAAGAC
PRP148	CCTCTCTTCAACTGCTCAATAATTTCCCGCTATGCAAAATTCCCCGTAC GCTGCAGGTCGAC
PRP149	GAAAGGAACTTTAGAAGTCCATTTCCTCGACCTTACCCTGGTTGATCG ATGAATTCGAGCTCG
PRP1194	AAGTCTGCTAATGGGAGACGAGGTTTTAGAAATTACCGAGGTCACTA CAGAGGAAGAAAGTCCGGTTCTGCTGCTAG
PRP1195	GAATGCTTGGCGAATGGTGTCGTAGTTATAAGTAACACTATTTATTTT TCTACTCTTCACCTCGAGGCCAGAAGAC
PRP131	AATCTTCAACATCAGGGCTATGTCAGGCCAGTTAGTTCAATGGAACG TACGCTGCAGGTCGAC
PRP132	GTATATATCATTGTCCGTAATATCATCGTGAAAACCAGTGTCCTCGAT CGATGAATTCGAGCTCG
PRP3438	GGATTCTAGAGACGCATTAGCTGAACATGCAGGAGCGCC
PRP3439	TCCCTTTCTATCAATTATGAGTTTATATATTTTTATAAT
PRP620	GATACAAAGGCTGATAGATGATTTCCTCAACATATCCGTTCTGAACTA TGATAATTACCACAGAATATGGCGTACGCTGCAGGTCGAC
PRP621	GTCCATGTTTCTATCGTACATAATTGTTTCGATCACATTCATCAACGCT ACTTCATTTTTTCTCATTAAAATCGATGAATTCGAGCTCG
PRP3434	AATCTTCAACATCAGGGCTATGTCAGGCCAGTTAGTTCAATGGAATG AAAGAGAGAGAGAGAAGC
PRP3435	GTATATATCATTGTCCGTAATATCATCGTGAAAACCAGTGTCCTCGGT CGTTCATGGTGACACTTT

PRP383	TAGGTATGATATCAGCACTAGATTCTATACCCGAGCCCCAAAACTCGTACGCTGCAGGTCGAC
PRP384	TCAGTAGTTGACTACGGGGAAGTCTTCTTCAGGCACTTGACAGTTATCGATGAATTCGAGCTCG

Supplementary Table S3. Oligonucleotides used for qPCR.

Name	Sequence (5’-3’)
ARO+	TGAGTCGTTACAAGGTGATGCC
ARO-	ACCTACAGGAGGACCCGAAA
DSB 0.2+	TCAGACTCAAGCAAACAATCAA
DSB 0.2-	CCCGTATAGCCAATTCGTTC
DSB 0.6+	CACCCAAGAAGGCGAATAAG
DSB 0.6-	CATGCGGTTCACATGACTTT
DSB 1.8+	ACGTCGTTGTTAATGGTGGTG
DSB 1.8-	CGCGAGTCTTATGCCAAAAA
DSB 5.4+	CGAGGAAAATGGTGGGATAA
DSB 5.4-	GGACGACTTTAAGATGGAAGGA

Supplementary Table S4. Summary of the main phenotypes relevant to the Tel1-Rad9 pathway.

Genotype/Condition	DNA damage resistance	DSB end-tethering	DSB resection	Checkpoint activation	Rad9 phenotype	Strand-invasion-dependent HR	SSA
wild type	wt	wt	wt	wt	wt recruitment and phosphorylation	wt	wt
tel1-400	wt	wt	wt	wt	wt recruitment; increased phosphorylation	wt	wt
sae2Δ	Reduced	Defective	Reduced	Persistent	Increased recruitment; increased phosphorylation	Reduced	Reduced
tel1-400 sae2Δ	Improved vs *sae2Δ* in a Rad9-dependent manner	Improved vs *sae2Δ* in a Rad9-dependent manner	Reduced	Persistent	Recruitment similar to *sae2Δ*; increased phosphorylation vs *sae2Δ*	Increased vs *sae2Δ*	Further reduced vs *sae2Δ*
rad9-7xA	Reduced at higher CPT concentration	Defective	wt	Defective	wt recruitment; impaired phosphorylation-dependent oligomerization	n.d.	n.d.
rad9-7xA sae2Δ	Improved vs *sae2Δ*	More defective vs *sae2Δ*	n.d.	n.d.	n.d.	NCO and CO similar to *sae2Δ*	Increased vs *sae2Δ*
tel1-400 rad9-7xA sae2Δ	No improvement vs *rad9-7xA sae2Δ*	No improvement vs *rad9-7xA sae2Δ*	n.d.	n.d.	n.d.	Reduced NCO and CO vs *tel1-400 sae2Δ*	Restored to *rad9-7xA sae2Δ* levels
rad9-ΔBRCT::FKBP	Reduced	Defective	n.d.	n.d.	Defective dimerization	n.d.	n.d.
rad9-ΔBRCT::FKBP + AP20187	Improved vs untreated	Improved vs untreated	n.d.	n.d.	Enforced dimerization	n.d.	n.d.
rad9-ΔBRCT::FKBP sae2Δ + AP20187	Improved to *sae2Δ* level	Improved to *sae2Δ* level	n.d.	n.d.	Enforced dimerization	n.d.	n.d.
tel1-400 rad9-ΔBRCT::FKBP sae2Δ + AP20187	Improved vs untreated; *tel1-400*-dependent rescue restored	Improved vs untreated; *tel1-400*-dependent rescue restored	n.d.	n.d.	Enforced dimerization	n.d.	n.d.

wt, wild type; n.d., not determined; HR, homologous recombination; NCO, non-crossover; CO, crossover; SSA, single-strand annealing.

Supplementary Figure S1

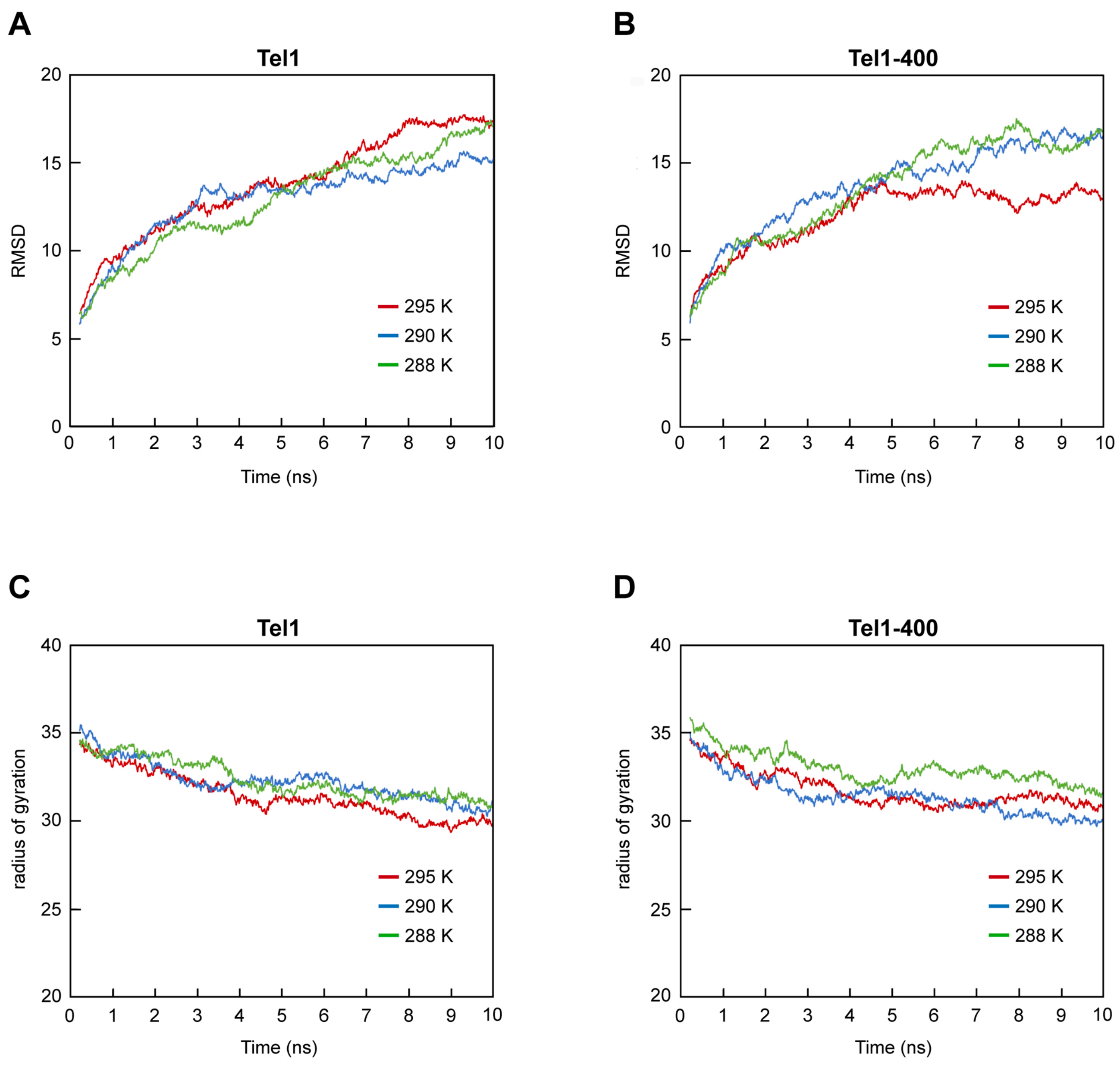

Supplementary Figure S1. CG-MD analysis of RMSD and radius of gyration. RMSD (**A,B**) and radius of gyration (**C,D**) calculated along the CG-MD trajectories of wild-type Tel1 (A and C) and Tel1-400 (B and D) at the indicated temperatures (288, 290, and 295 K).

Supplementary Figure S2

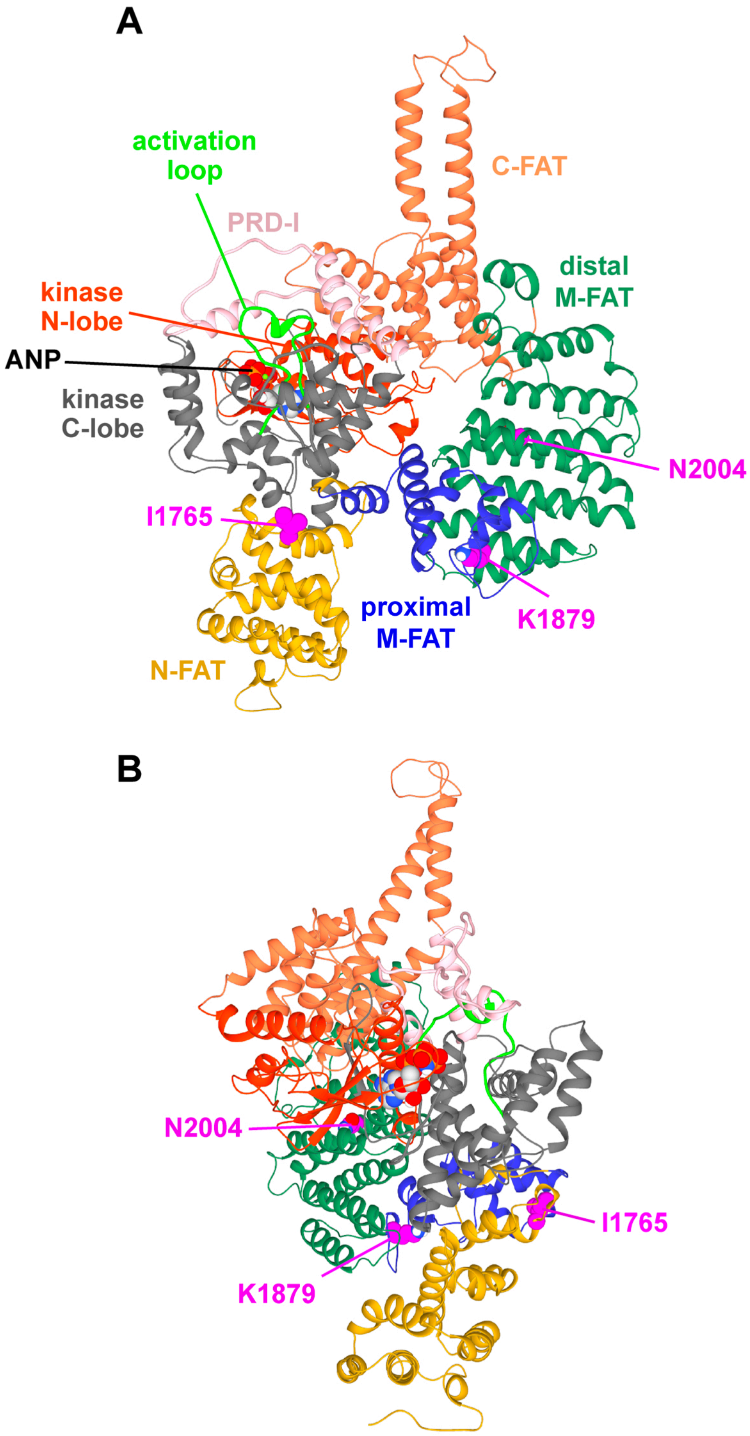

Supplementary Figure S2. Tel1 kinase domain activation requires a conformational change in the C-FAT and kinase N-lobe domains. **(A,B)** A subunit from the inactive dimeric Tel1 structure (PDB ID 6S8F) is shown. **(C,D)** A subunit from the active-like asymmetric Tel1 conformation (a complete model based on PDB ID 6JXA) is shown. In (B) and (D), the views are rotated by 120° around the Y axis relative to (A) and (C), respectively. The FAT domain is divided into three subregions: N-FAT (residues 1601-1786; yellow ochre); M-FAT, which is subdivided into a proximal segment (residues 1787-1870; blue) and a distal segment (residues 1871-2128; green); and C-FAT, comprising the LBE-interacting domain (LID) and the HEAT-repeat domain (HRD) (residues 2129-2355; coral). The kinase domain comprises the N-lobe (residues 2356-2509; reddish orange) and the C-lobe (residues 2510-2787; grey). The PRD-I loop (residues 2696-2737; pink) is fully disordered in the asymmetric Tel1 structure shown in (C) and (D) [37]. The activation loop is shown in bright green. The ATP analog ANP (adenosine 5′-[β,γ-imido]triphosphate) is shown as spheres, with oxygen in red, nitrogen in blue, hydrogen in white, phosphorus in orange, and Mg^{2+} as a green sphere. Mutated residues are shown as magenta spheres.

Supplementary Figure S3

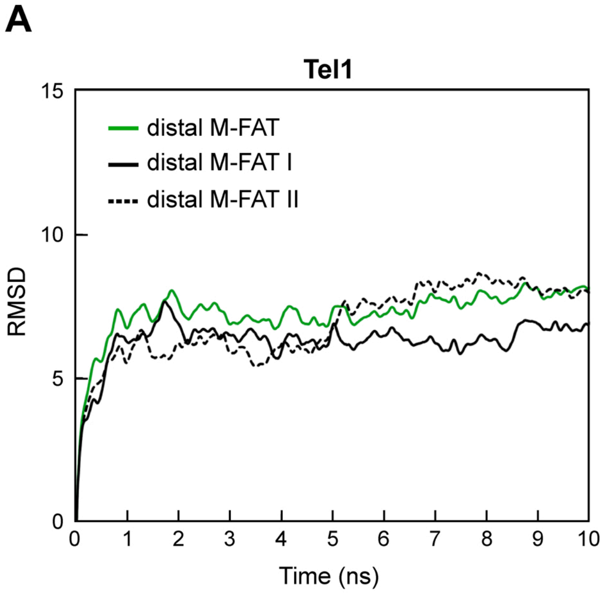

Supplementary Figure S3. Altered dynamic properties of the distal M-FAT in Tel1-400. RMSD values calculated along the CG-MD trajectories at 290 K for the indicated distal M-FAT substructures in wild-type Tel1 (**A**) and Tel1-400 (**B**), using models spanning residues 1601-2787. Distal M-FAT I encompasses residues 1880-1986, whereas distal M-FAT II spans residues 1987-2128.

Supplementary Figure S4

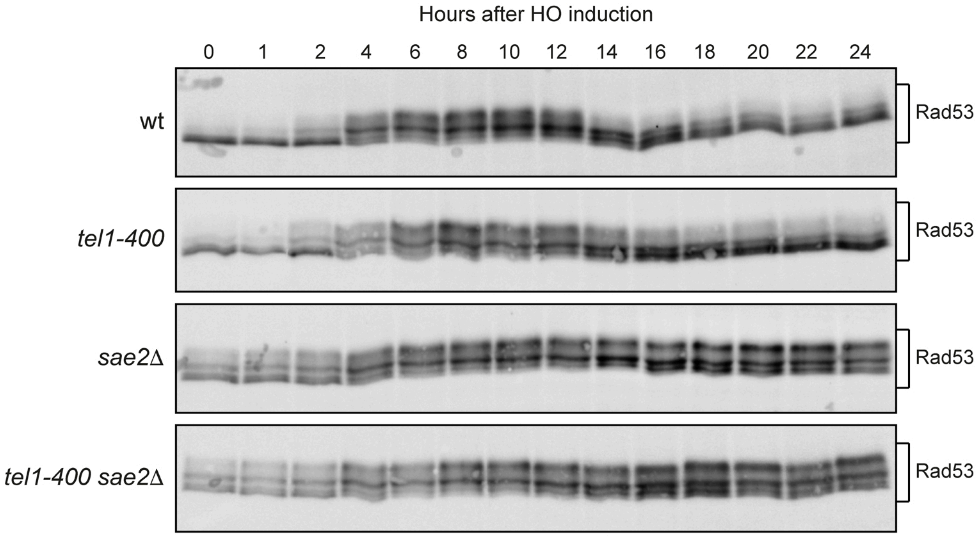

Supplementary Figure S4. Rad53 phosphorylation following induction of an irreparable HO-induced DSB. Exponentially growing cells in YEPR were shifted to YEPRG (time zero) to induce HO expression, and Rad53 phosphorylation was monitored by western blot using an anti-Rad53 antibody.

Supplementary Figure S5

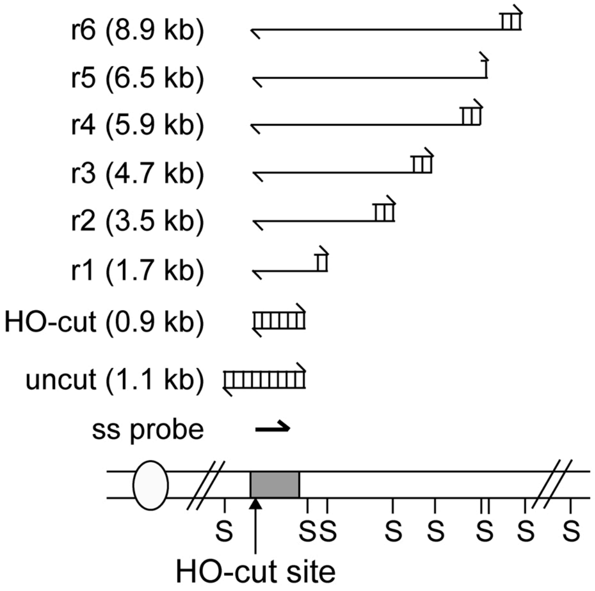

Supplementary Figure S5. System used to detect DSB resection. Resection of DSB ends progressively eliminates *Ssp*I sites (S), generating increasingly long *Ssp*I fragments (r1-r6) that can be separated on an alkaline agarose gel and detected by hybridization with a strand-specific RNA probe that anneals to the unresected strand on one side of the break.

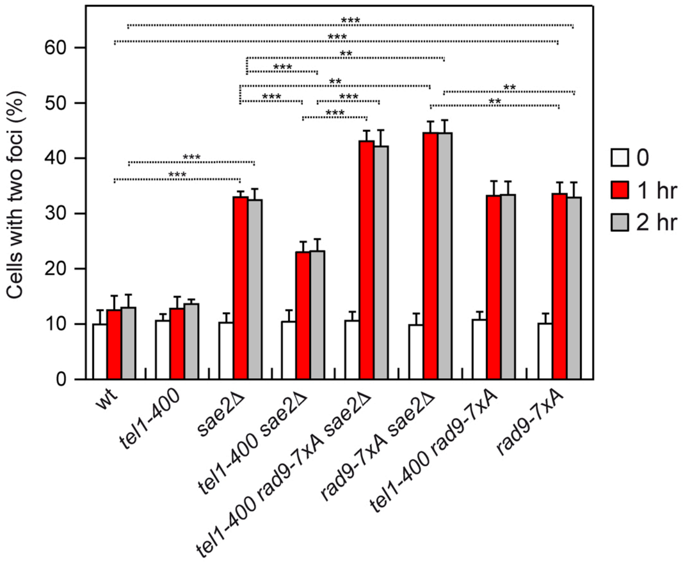

Supplementary Figure S6. DSB end-tethering assay in G2-arrested cells. Exponentially growing cells in YEPR were arrested in G2 with nocodazole (time zero) and shifted to YEPRG in the continuous presence of nocodazole. For each strain, 200 cells were scored to determine the percentage of cells displaying two LacI-GFP foci. Bars represent the mean ± s.d. from three independent experiments. ****p*<0.005; ***p*<0.01 (unpaired two-tailed Student's *t*-test).

Supplementary Figure S7

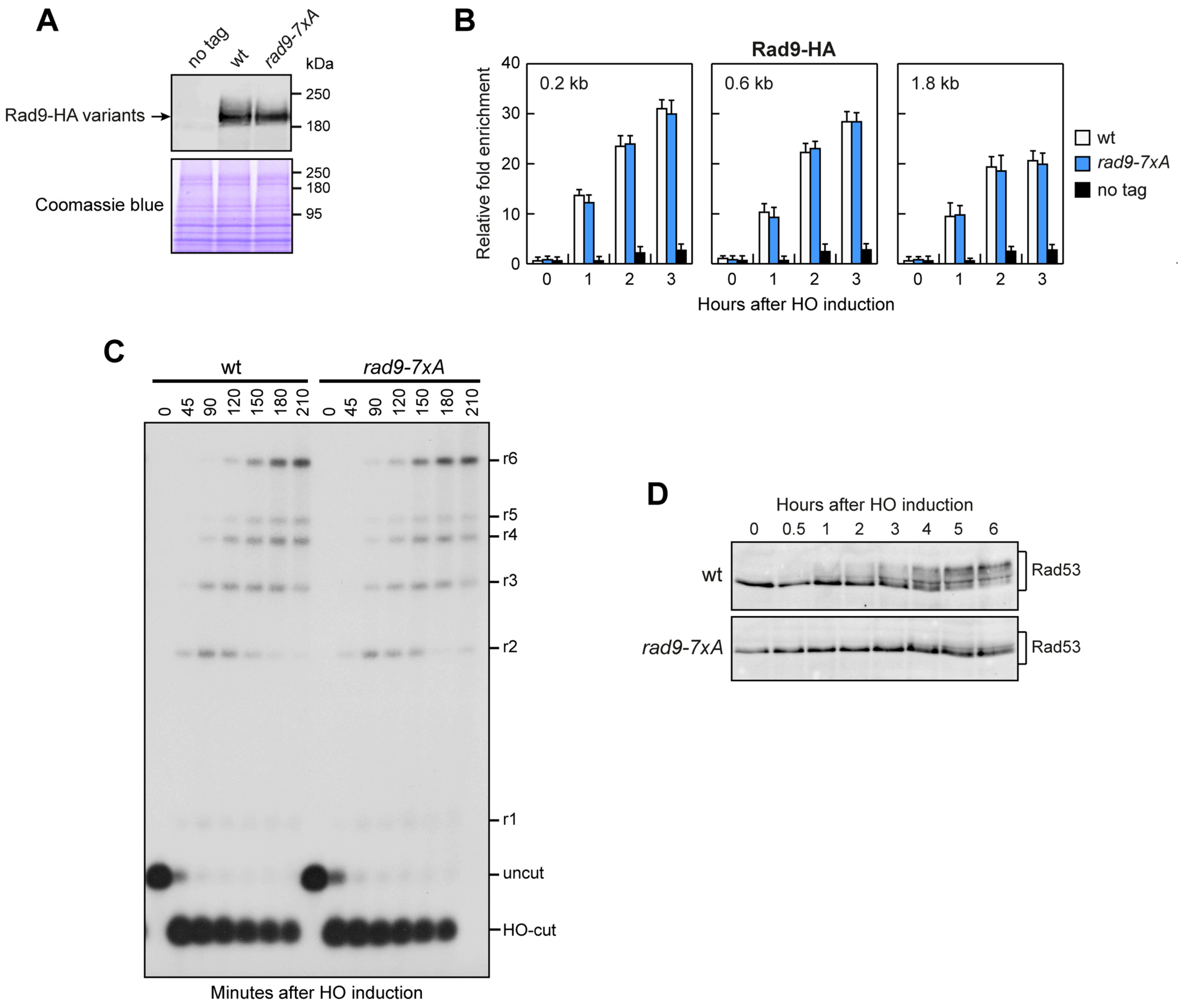

Supplementary Figure S7. Rad9 association, DSB resection, and checkpoint activation in *rad9-7xA* cells. (**A**) Protein extracts from exponentially growing cells were analyzed by western blot using an anti-HA antibody. (**B**) ChIP-qPCR analysis of Rad9 occupancy at an HO-induced DSB. Relative Rad9-HA enrichment at the indicated distance from the HO cut site was measured by anti-HA ChIP followed by qPCR. Bars represent mean ± s.d. from three independent experiments. (**C**) DSB resection assay. Exponentially growing YEPR cultures of JKM139 derivative strains were shifted to yeast extract, peptone, raffinose and galactose (YEPRG) at time zero to induce HO. *Ssp*I-digested genomic DNA was separated on an alkaline agarose gel and hybridized with a strand-specific *MAT* probe complementary to the unresected strand. Progressive 5'-3' resection eliminates *Ssp*I sites, generating longer *Ssp*I fragments (r1-r6) detected by the probe. (**D**) Rad53 phosphorylation following induction of an irreparable HO-induced DSB. Exponentially growing cells in YEPR were shifted to YEPRG at time zero to induce HO, and Rad53 phosphorylation was monitored by western blot using an anti-Rad53 antibody.

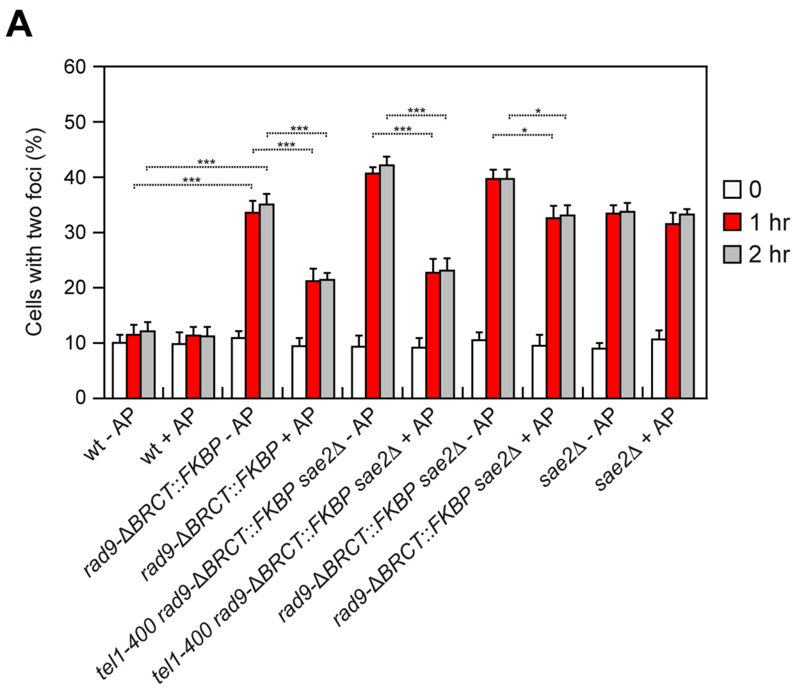

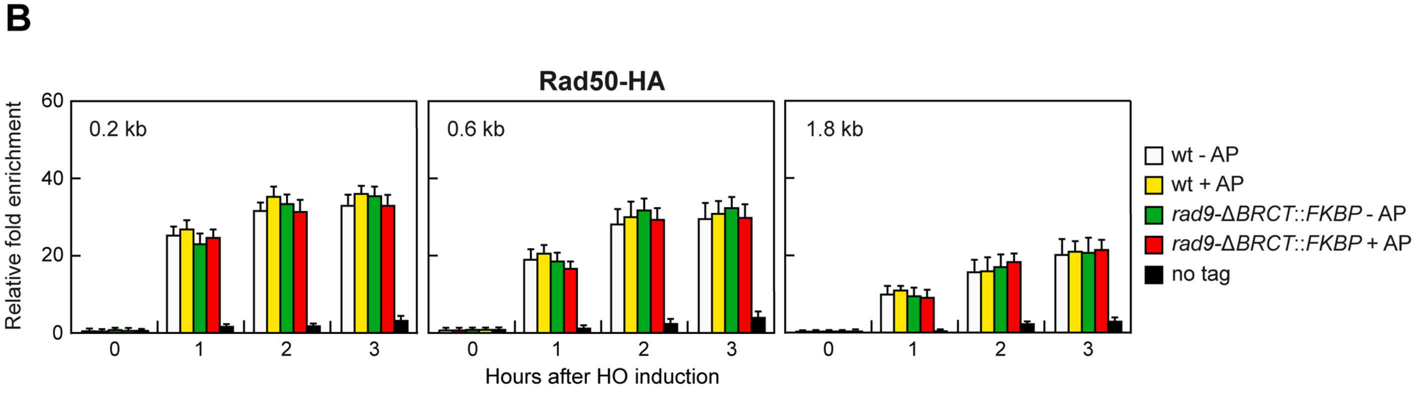

Supplementary Figure S8. DSB end-tethering assay in G2-arrested cells and Rad50 occupancy at an HO-induced DSB. (**A**) Exponentially growing cells in YEPR were arrested in G2 with nocodazole (time zero) and shifted to YEPRG in the continuous presence of nocodazole. For each strain, 200 cells were scored to determine the percentage of cells displaying two LacI-GFP foci. Bars represent the mean ± s.d. from three independent experiments. ****p*<0.005; **p*<0.05 (unpaired two-tailed Student's *t*-test). (**B**) Relative Rad50-HA enrichment at the indicated distance from the HO cut site was measured by anti-HA ChIP followed by qPCR. Bars repersent the mean ± s.d. from three independent experiments.

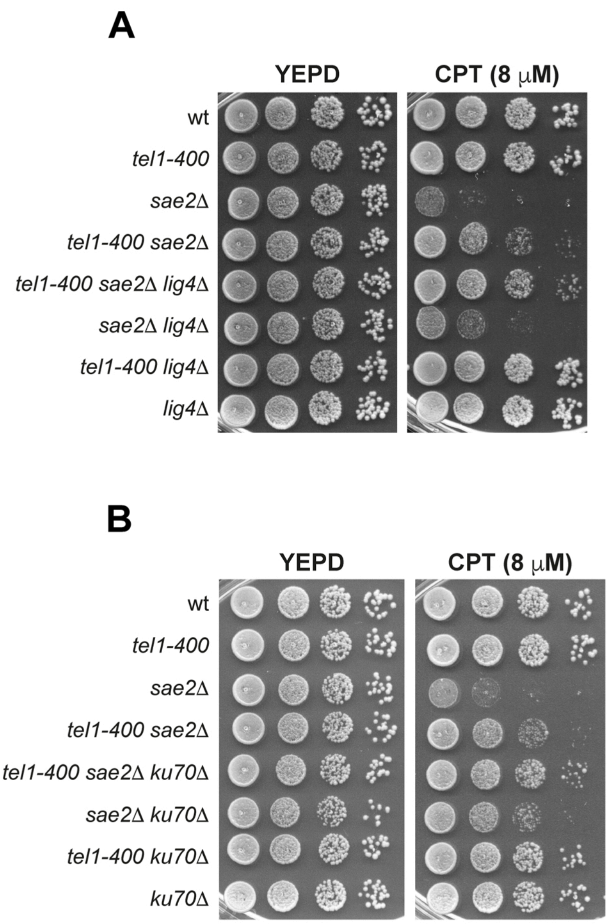

Supplementary Figure S9. Drug-sensitivity assay. (**A,B**) Exponentially growing cultures were serially diluted (1:10) and spotted onto YEPD plates with or without CPT.

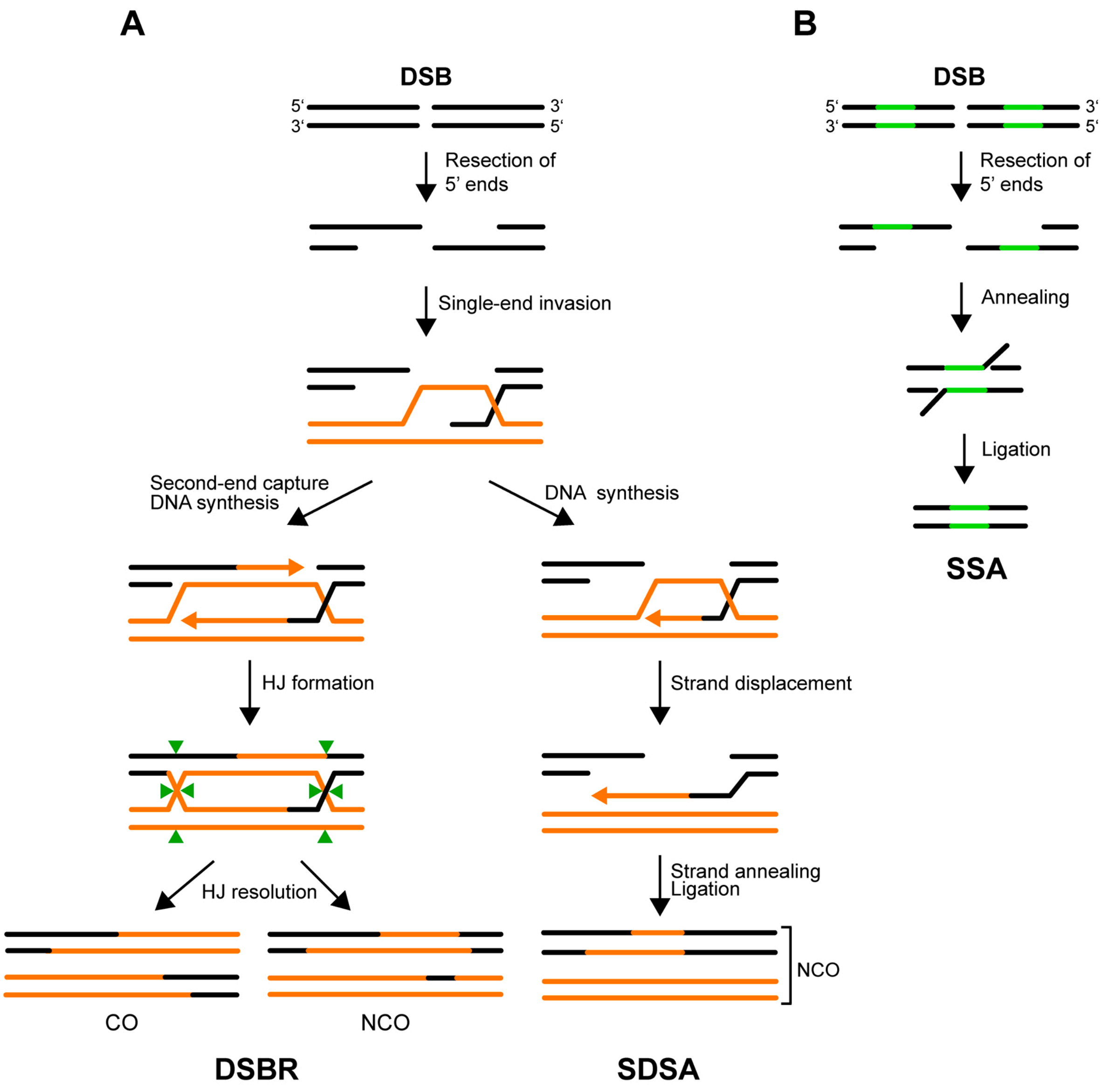

Supplementary Figure S10. Models of homology-dependent recombination pathways. (**A**) In the canonical double-strand break repair (DSBR) model (left), capture of the second ssDNA end, DNA synthesis, and ligation generate a double Holliday junction (HJ). Resolution of both junctions in the same orientation, either horizontal or vertical, produces a non-crossover (NCO) product, whereas resolution in different orientations generates a crossover (CO) product. In the synthesis-dependent strand annealing (SDSA) model (right), the newly synthesized strand is displaced and anneals to the 3′ ssDNA tail at the other end of the DSB, after which DNA synthesis completes repair. (**B**) When a DSB occurs between direct repeats (green lines) and resection proceeds sufficiently to expose the duplicated sequences, the resulting complementary ssDNA regions anneal, leading to deletion of one repeat and the intervening sequence through a mechanism known as single-strand annealing (SSA). Lines represent individual DNA strands, and arrowheads indicate the 3′ ends of newly synthesized strands.

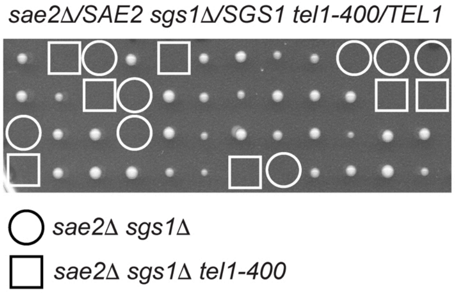

Supplementary Figure S11. Combined deletion of *SGS1* and *SAE2* is synthetically lethal in both the presence and absence of the *tel1-400* allele. Meiotic tetrads were dissected on YEPD plates and incubated at 25°C, after which the resulting spores were genotyped.

Supplementary Movie Legends

Supplementary Movie S1. Movie showing the CG-MD trajectory of wild-type Tel1 (residues 1601-2787) at 290 K. N-FAT (residues 1601-1786; yellow ochre); M-FAT, which is subdivided into a proximal segment (residues 1787-1870; blue) and a distal segment (residues 1871-2128; green); C-FAT (LID/HRD; residues 2129-2355; coral); the kinase N-lobe (residues 2356-2509; orange); the kinase C-lobe (residues 2510-2787; grey).

Supplementary Movie S2. Movie showing the CG-MD trajectory of Tel1-400 (residues 1601-2787) at 290 K. N-FAT (residues 1601-1786; yellow ochre); M-FAT, which is subdivided into a proximal segment (residues 1787-1870; blue) and a distal segment (residues 1871-2128; green); C-FAT (LID/HRD; residues 2129-2355; coral); the kinase N-lobe (residues 2356-2509; orange); the kinase C-lobe (residues 2510-2787; grey).
